# Microbiome–Epigenome Interplay Impacts Microbial Symbiosis and Stress Adaptations in the Brown Planthopper (*Nilaparvata lugens*)

**DOI:** 10.3390/ijms27167357

**Published:** 2026-08-17

**Authors:** Ayushi Gupta, Suresh Nair

**Affiliations:** Plant-Insect Interaction Group, International Centre for Genetic Engineering and Biotechnology (ICGEB), Aruna Asaf Ali Marg, New Delhi 110067, India; ayushi@icgeb.res.in

**Keywords:** DNA methylation, antibiotics, *Nilaparvata lugens*, *16S rRNA*, insect evolution

## Abstract

The gut microbiota and epigenetic processes both contribute to insect survival and adaptation; however, the relationship between these two systems remains largely unexplored. In this study, we used the brown planthopper (*Nilaparvata lugens*; BPH) to explore microbiome–epigenome interactions and evaluate its impact on BPH survivability under environmental stress. Disruption of the gut microbiome using antibiotics significantly altered the epigenetic profile of various stress-responsive genes in the BPH. Similarly, perturbations in the epigenome induced by 5-azacytidine resulted in an altered microbiome with diverse metabolic capacities, thus indicating the potential role of epigenetics in maintaining microbial symbiosis in BPH. Further, analysis of gene expression profiles revealed that 5-azacytidine treatment altered the mRNA levels of various genes involved in BPH immunity, suggesting that epigenetic mechanisms regulate and sustain microbial symbionts in insects by modulating their immune system. Altogether, these findings suggest an interplay between the epigenome and microbiome, that influences gene regulation and microbe-mediated regulation of shared metabolic pathways in BPH. Our results highlight new research avenues into the molecular mechanisms of symbiont-enabled herbivory and have implications for future studies on the relationship between gut microbiota and epigenetic mechanisms, the evolution of these processes and their effects on insect–plant interactions in changing environments.

## 1. Introduction

Insects regularly encounter environmental fluctuations and stressors, but survive due to their rapid adaptive nature. Epigenetic reprogramming is one mechanism that facilitates rapid adaptations in insects [1]. However, these epigenetic modifications can sometimes be deleterious. Under such circumstances, the gut microbiome provides insects with a flexible alternative for adaptation without compromising survival. Insects maintain close associations with their highly dynamic gut microflora, which, via continual shifts in its composition and function, influences their fitness, longevity and stress responses [2,3]. This suggests the existence of molecular mechanism(s) that allows insects to sense external stimuli and quickly alter their gut microflora. Considering the responsiveness of DNA methyltransferases (DNMTs), it is likely that DNA methylation mediates insect–microbiome interactions during adaptation [4]. This bidirectional interaction is increasingly recognised as an important mechanism underlying adaptations in diverse organisms.

Studies indicate that insect epigenomes shape microbial associations by regulating immune genes through DNA methylation and other epigenetic mechanisms [5,6,7,8]. Conversely, bacterial symbionts can also influence host DNA methylation patterns. Evidence from vertebrates demonstrates that microbes can induce epigenetic modifications in the host immune genes to maintain homeostasis [9]. Whether comparable microbiome–epigenome interactions occur in insects, however, remains largely unexplored.

Host metabolism is considered one of the principal interfaces linking the microbiome with the epigenome [10]. Bacterial endosymbionts can influence DNA methylation within insects by (i) directly impacting the enzymes involved in epigenetic modifications and/or (ii) altering the substrate levels available for these enzymes. Several microbial-derived metabolites such as folate, acetate, butyrate, propionate, and S-adenosyl methionine can directly impact the host’s epigenetic profile by modulating DNMTs and related enzymes [11,12,13,14,15,16,17]. However, specific bacterial species, the signalling pathways, and the transcriptional machinery targeted in the host remain unknown. Further, gut microbes can also modify host responses to environmental stresses by altering the epigenome and affecting gene expression [10,17]. Several microbe-derived bioactive compounds and small RNAs regulate the expression of crucial symbiotic genes and manage fundamental housekeeping processes in their insect hosts through epigenetic pathways [18,19,20,21]. Interestingly, insect-derived microRNAs can also regulate microbiome function in higher organisms [22], although this phenomenon is poorly characterised in insects.

Our understanding of microbiome and epigenome crosstalk in insects remains fragmentary with most insights derived from studies on vertebrates or individual insect symbionts, particularly *Wolbachia*. In mosquitoes, the pathogenic *Wolbachia* (strain *w*MelPop) suppresses *DNMT2*, thereby altering host DNA methylation [23,24]; one of the few direct demonstrations of microbial modulation of the insect epigenome. However, whether such interactions are widespread across other insect species or involve complex gut microbial communities is unknown. Equally unresolved is the reciprocal relationship, i.e., whether epigenetic perturbations influence microbial community composition and function. Addressing this gap is essential for understanding insect symbiosis, adaptation, and evolution.

Guided by this perspective, this study uses the brown planthopper (*Nilaparvata lugens*; BPH) to explore microbiome–epigenome interactions. As a Hemipteran insect with robust DNA methylation machinery [25], BPH is an ideal model. Previous studies have demonstrated that both epigenetic regulation and gut microbiota influence its stress tolerance, survival, and ecological adaptation [1,26,27,28,29,30,31]. But whether these two regulatory systems interact remains unknown. To elucidate this, we tested two approaches, i.e., (i) the impact of antibiotic-mediated disruption of gut microbiome on epigenetic profiles of stress-responsive genes in the host, and (ii) the effect of pharmacological inhibition of DNA methylation (via 5-azacytidine) on gut microbial community structure. Together, results from these analyses establish a foundation for understanding bidirectional microbiome–epigenome interplay in insect adaptation.

## 2. Results

### 2.1. Antibiotic Treatment

#### 2.1.1. Antibiotic Treatment and Its Impact on Global DNA Methylation in BPH

**Figure 1:**
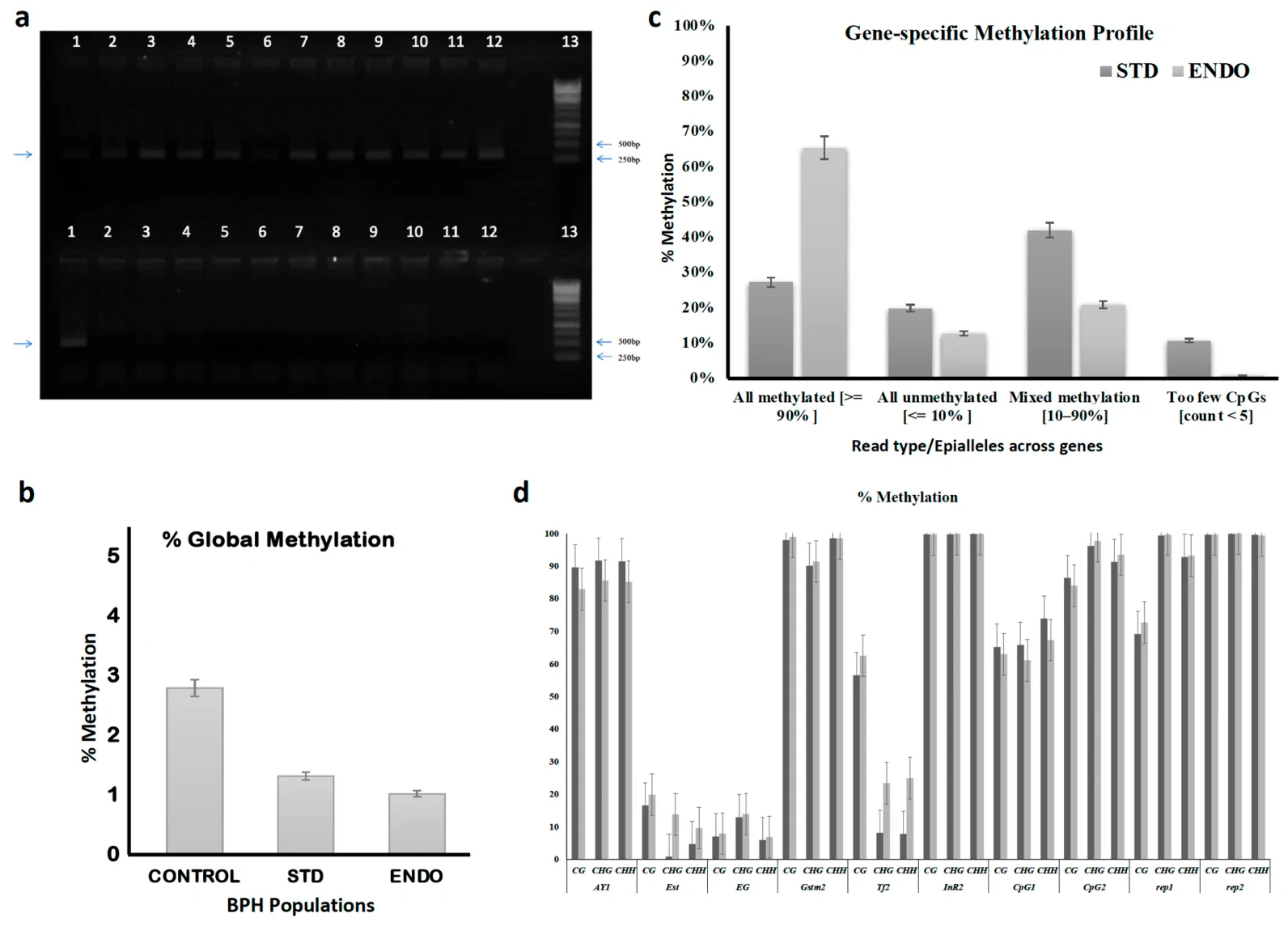
Effect of antibiotics on the microbiome and methylation status of the BPH. (**a**) Effect of antibiotic treatment (48 h) on bacterial titres in BPH individuals as revealed by semi-quantitative PCR assay performed using primers targeting the V3-V4 region of *16S rRNA* (for details see “Materials and Methods”). Lane 1: BOD insects (untreated); Lanes 2–12: Amplification of the *16S rRNA* fragment from BPH individuals exposed to antibiotics; Lane 13: 1 Kb ladder. The upper panel represents PCR amplification controls (*actin*; see “Materials and Methods” for details) for the respective lanes on the lower panel (*16S rRNA* fragment). Arrows on the left indicate the PCR amplified fragments (283 bp *actin* fragment and 483 bp *16S rRNA* fragment). Arrows on the right indicate the 500 and 250 bp fragments of the 1 Kb ladder. (**b**) Comparison of global DNA methylation profiles of BPH individuals treated with antibiotics (ENDO) with those fed only on standard media (STD; without antibiotics) and control population (CONTROL; BPH individuals fed on TN1 rice variety). Error bars represent ± SD. Labels on the X-axis indicate epialleles antibiotic-treated (ENDO) and untreated (STD) samples and numbers on the Y-axis represent percent methylation. (**c**) Total percent methylation detected across the stress-responsive genes with their distribution profiles in antibiotic-treated (ENDO) and untreated (STD) samples. Error bars represent ± SD. Labels on the X-axis indicate samples and numbers on the Y-axis represent percent methylation. (**d**) Variations in percent methylation across stress-responsive genes (in CG, CHG and CHH context) upon exposure to antibiotic-treated (ENDO) and untreated (STD) samples of BPH. Error bars represent ± SD. Labels on the X-axis indicate gene names and numbers on the Y-axis represent percent methylation.

Quantification of bacterial load after 48 h of antibiotic exposure revealed approximately > 98% reduction in bacterial titres in BPH compared to the unexposed individuals (Figure 1a), which confirmed the efficiency and reliability of the treatment. The treated insects with minimal microflora were thus considered suitable for methylation analyses. The antibiotic-mediated disruption of the microbiome resulted in changes in the global DNA methylation levels. The amount of 5-methylcytosine in the antibiotic-treated insects reduced from 1.32% (i.e., in BPH fed on standard media without antibiotics) to 1.025% (i.e., in insects fed on standard media supplemented with antibiotics). Although the antibiotic-induced alteration in the global DNA methylation level was minimal, a remarkable difference (hypomethylation) was observed when insects were transferred from the rice plants (TN1) to an artificial diet (i.e., methylation levels reduced from 2.8% to 1.32%; Figure 1b), implying that dietary changes induce a much more profound response (hypomethylation) at the whole-genome level.

#### 2.1.2. Antibiotic Treatment Resulted in Hypermethylation of Stress-Responsive Genes in BPH

In the present study, we performed targeted bisulfite sequencing to screen for antibiotic-induced de-methylation/methylation marks across the predicted CpG islands at a single-nucleotide resolution (see the “Materials and Methods” section). This process provided a cost-effective and rapid method for screening for antibiotic-induced epigenetic changes, particularly when focusing on CpG islands, thereby enabling one to observe the changes in specific genomic regions of interest with significantly higher data quality than with whole-genome approaches. In this regard, the bisulfite conversion efficiency was reliable and estimated to be >99%.

After bisulfite treatment, sequencing libraries yielded > 0.4 million reads with more than 98% exhibiting high quality (Q value > 30). The mapping efficiency was 51.7% for antibiotic-treated samples (i.e., ENDO) and 30.4% for STD samples (Table S1). The mapping efficiency can be influenced by factors such as bisulfite-induced sequence complexity reduction, genomic characteristics, and the targeted nature of the sequencing approach. Despite these differences, all samples satisfied the quality control criteria established for downstream analysis, and the analysed loci exhibited sufficient sequencing depth to enable reliable methylation calling.

Further, methylation calling data (in all three contexts) revealed differential methylation across genes and between treatments (Figure 1c). After antibiotic treatment, we observed an overall increase in the percentage of uniformly methylated alleles, i.e., from 27.25% (for STD) to 65.41% (for ENDO), and a reduction in uniformly unmethylated alleles, i.e., from 19.91% (STD) to 12.77% (ENDO). In addition, the percentage of alleles with mixed methylation patterns (i.e., methylation restricted to a few cytosines within the CpG island) decreased from 42.07% to 20.93% (Figure 1c).

Additionally, methylation calling data in all three contexts revealed that, while antibiotic treatment caused an overall dip in the methylation status across CpG islands corresponding to *Esterase*, *Endoglucanase*, *Gsmt2*, and *Tf2*, the methylation values increased for *CYP6AY1*, *CYP6ER1*, and *rep1*. In addition, no change was observed for *InR2* and *rep2* (Figure 1d). These observations were in consonance with the results obtained from the site-specific analysis of methylation status across each gene (Figure S1). Next, as methylation percentage by itself is not reliable for determining the actual coverage of the site, methylation count data for each cytosine and its coverage were statistically validated. Here, the differentially methylated cytosines (DMCs) between treatments corresponding to *CYP6ER1*, *Esterase*, *Gstm2*, *Tf2*, and *rep1* were identified (Table S2). Collectively, the results suggest that disruption of the microbiome directly or indirectly affected the epigenetic profile of the insect host. Likewise, to study whether epigenetic processes influence the gut microbiome, we disrupted the epigenetic profile of BPH using azacytidine and studied its impact on the microbiome.

### 2.2. Azacytidine Treatment

#### 2.2.1. Azacytidine Treatment Caused Inhibition of DNMTs and Subsequent Hypomethylation Across BPH Genes

**Figure 2:**
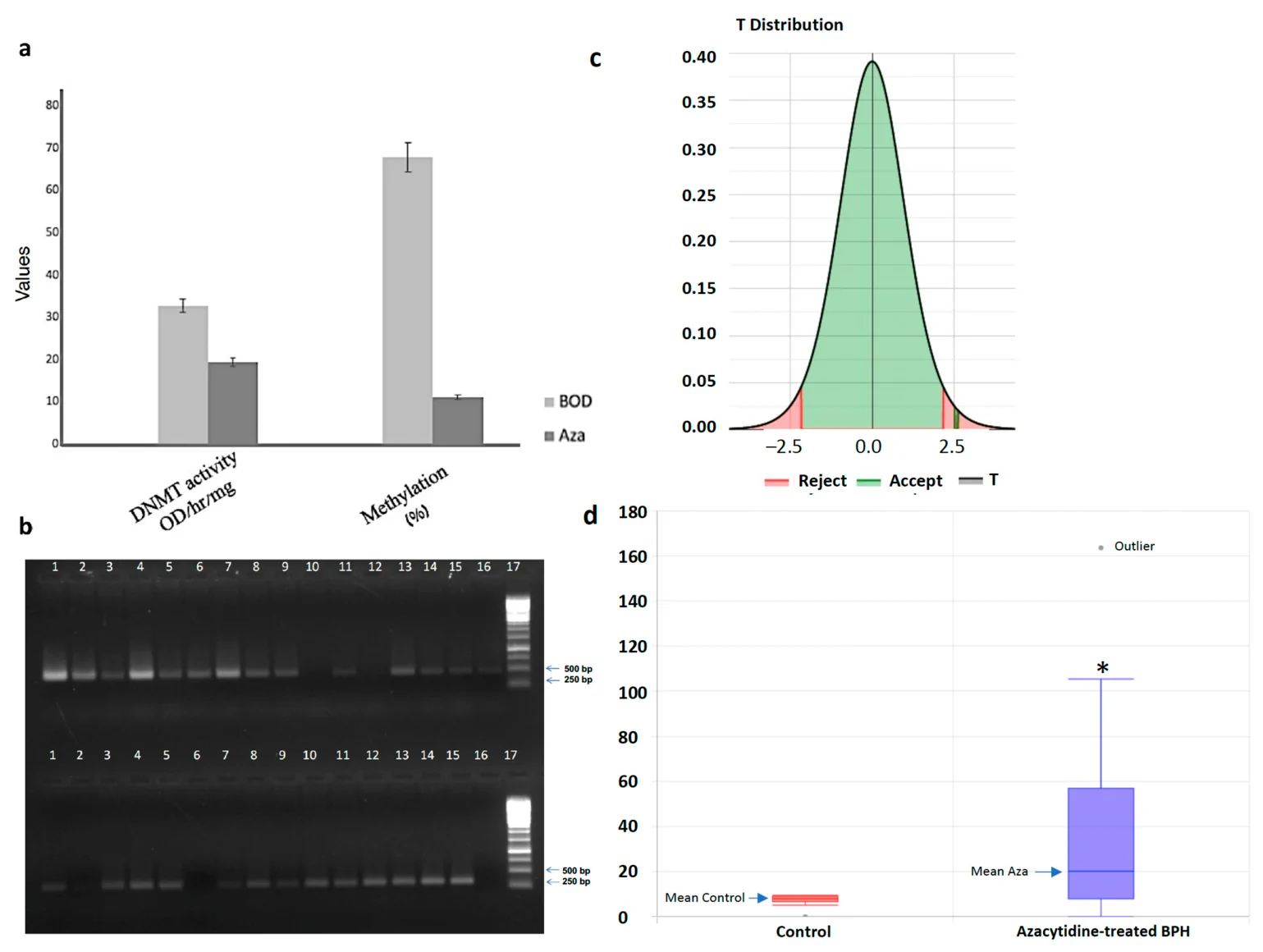
5-azacytidine-mediated disruption of BPH methylome led to increased bacterial titres in BPH. (**a**) 5-aza-mediated reduction in cellular DNMT levels (OD/hr/mg) and percent methylation (%) across stress-responsive genes in BPH. Error bars represent ± SD. (**b**) Individuals from 5-aza-treated and untreated (control) BPH populations were assayed by semi-quantitative PCR using *16S rRNA* primers. Samples assayed were BPH individuals from populations that were: (I) exposed to 5-aza for 48 h (lanes 1–8) and (II) unexposed to 5-aza (lanes 9–16); 1 Kb DNA ladder (Lane 17). The semi-quantitative PCR products were separated on a 1.0% agarose gel. The top panel of the gel represents amplification of the *16S rRNA* fragment (483 bp) from BPH individuals and the lower panel represents PCR amplification controls (*actin*) for the respective lanes on the upper panel. Arrows on the right indicate the 500 and 250 bp fragments of the 1 Kb ladder. (**c**) *t*-test distribution graph (*p* ≤ 0.05; *N* = 48) for the estimated bacterial titres in each group (5-aza-treated and control). (**d**) Box plots indicating a significant (*) increase in the bacterial load in BPH following 5-aza treatment. Boxes represent interquartile range and whiskers span all data points. Mean values, standard deviation and outliers are indicated. Note: samples which failed to amplify the *actin* fragment were not included for determining the bacterial load.

Before assessing the effect of azacytidine on the BPH microbiome, we confirmed whether azacytidine exposure for 48 h has indeed disrupted the methylation profile of the BPH genome. In this context, DNMT activity assays revealed an approximate 40% reduction in DNMT activity following 5-aza treatment (Figure 2a). To further validate the efficiency and reliability of the treatment, a methylation-sensitive restriction assay was performed. This method is efficient for evaluating CpG methylation at specific loci. The results demonstrated a marked decline in methylation levels (i.e., from 70% to 12%) across BPH genes following 5-aza treatment (Figure 2a). Hence, given its potent and effective inhibition of DNA methylation, it was used to study the impact of a disrupted epigenome on the insect’s microbiome.

#### 2.2.2. 5-Aza Treatment Led to Increased Microbial Titres in BPH

A substantial increase (approximately >5-fold) in the bacterial load following 48 h 5-aza treatment was observed in BPH as estimated by the semi-quantitative PCR assay. In addition, Student’s *t*-test (*p*-value cut-off < 0.05) deemed the differences observed between bacterial titres across the two groups (i.e., treated and untreated insects) significant (T-statistics 2.56, effect size 0.68; Figure 2b–d). Further, *16S rRNA* amplicon metagenomic sequencing was performed to study the microbial profile of 5-aza-treated and untreated samples.

#### 2.2.3. Alterations in Bacterial Diversity and Abundance Observed in BPH Following 5-Aza Treatment

*16S rRNA* amplicon metagenomic sequencing for 5-aza-treated and untreated (BOD; lab grown population of BPH) samples generated 9,15,794 reads on the Illumina NovaSeq platform. Of these, 8,10,636 were of high quality and hence were retained after base calling, quality filtering, and adapter trimming for downstream analyses. Subsequently, 7,94,015 paired-end (PE) reads were successfully merged and contigs formed, subjected to quality filtering and chimaera removal. Thereafter, 6,28,606 reads were regarded as Effective Clean Tags and were used for mapping and taxonomic assignment (Table S3).

**Figure 3:**
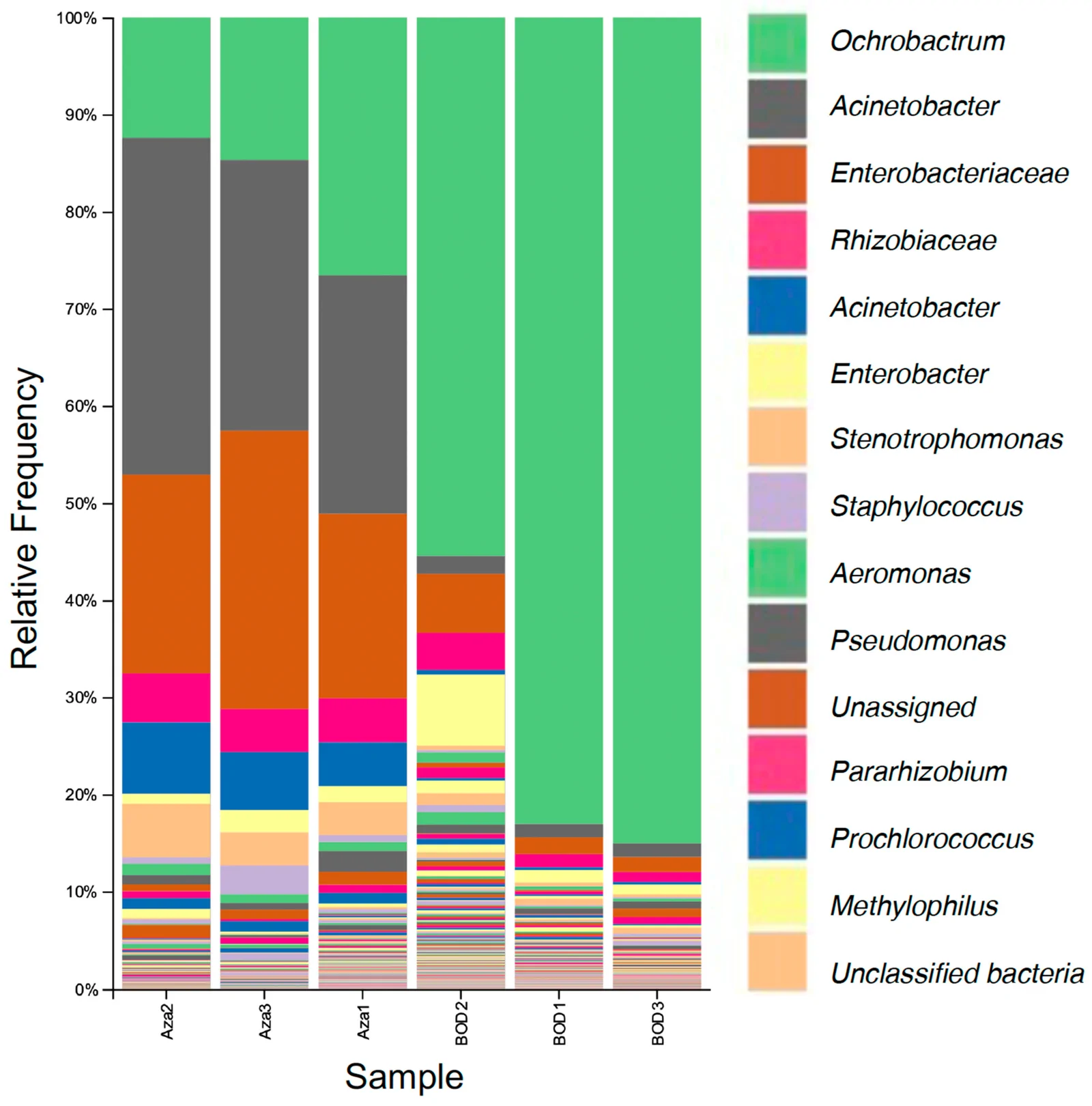
Overview of biodiversity of the bacterial kingdom in the control samples compared with the 5-aza-treated samples. Stacked bar plots depicting the variations in microbial composition, their relative abundance and diversity between 5-aza-treated (Aza1 to Aza3) and untreated (BOD1 to BOD3) samples. The top 15 most abundant bacterial taxa are shown.

**Figure 4:**
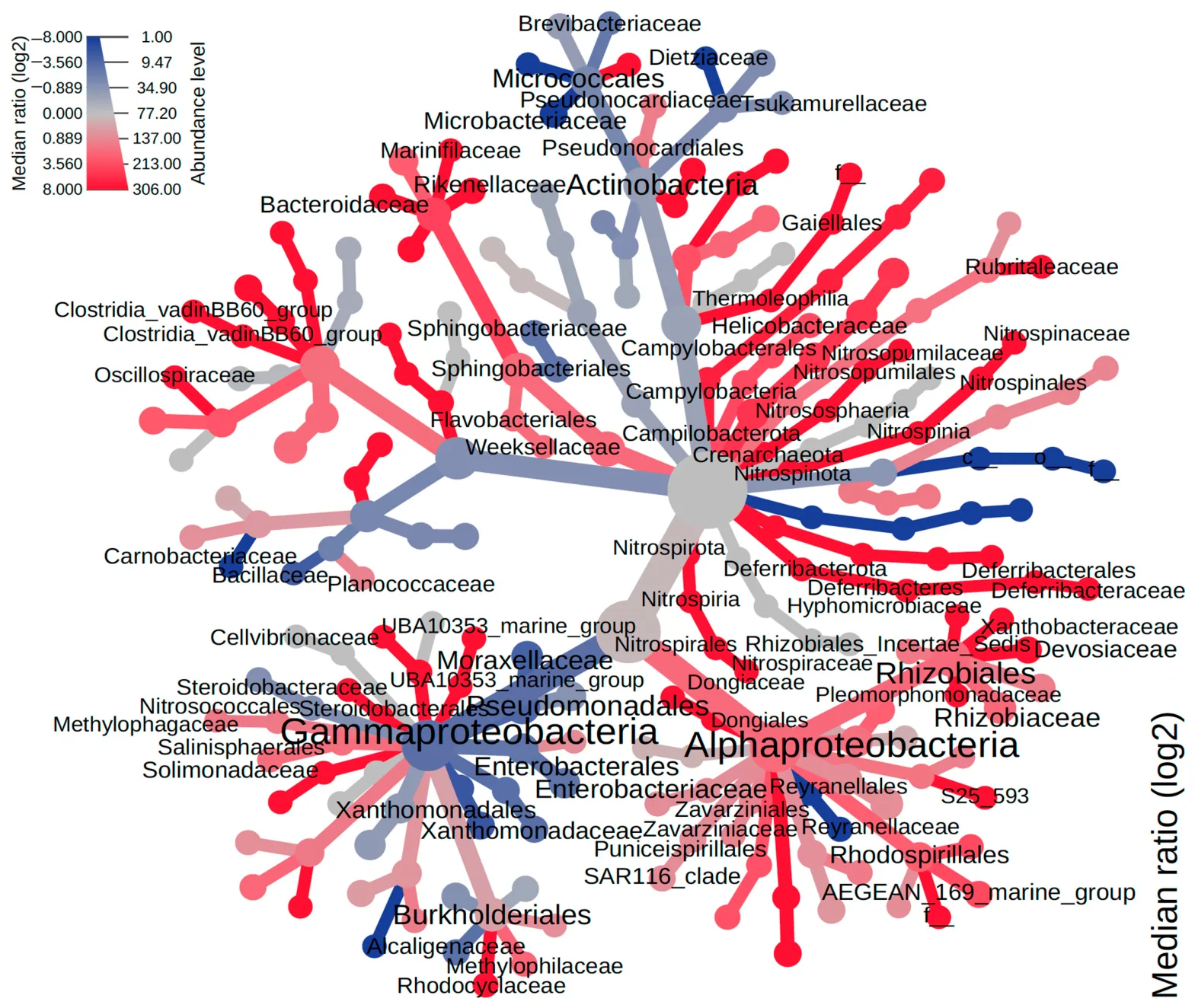
Differential heat tree depicting the change in taxa abundance between 5-aza-treated (Aza) and untreated (BOD) samples at family level. The size and colour of nodes and edges are correlated with the abundance of taxa. The size of the individual nodes in the cladogram depicts the number of taxa identified at that taxonomic level. The taxa identified as differently represented are according to the Wilcoxon statistical test. The legend to the nodes can be read as follows: left side, “Log2 ratio” of the median proportions of the taxa corresponding to the colour scale, right side, “Number of ASVs” where the diameter reflects the number of ASVs corresponding to the nodes on the heat trees. The branches with differential abundance are coloured according to their respective categories shown for each tree. The colour gradient and the size of the node, edge, and label are based on the log2 ratio of median abundance.

Post-taxonomic classification, an average of 95,865 ASVs were identified in BOD (untreated) samples, while only 73,351 were detected in 5-aza-treated insects (Table S4). Rarefaction curve analysis confirmed sufficient sequencing depth and comprehensive coverage supporting the reliability of the dataset (Figure S2). However, the data were normalised to eliminate uneven sequencing depths. And based on the annotation of ASVs and the feature counts thus obtained for each sample, the species abundance values at different taxonomic levels (i.e., kingdom, phyla, class, order, family, genus, and species) were obtained (Table S3). The relative abundance plot generated to view the bacterial composition and their proportions in each sample revealed marked differences in community structure, relative abundance and overall diversity between 5-aza-treated and untreated (BOD) samples (Figure 3). It was observed that the exposure to 5-aza increased the titres of various pathogenic bacteria such as *Acinetobacter*, *Enterobacter*, *Stenotrophomonas*, *Reyranella*, and *Staphylococcus* while simultaneously decreasing the amounts of *Ochrobactrum*, *Methylophilus*, and *Elizabethkingia* (Figure 4 and Figure S3).

Further, alpha and beta diversity analyses were performed to assess the microbial community diversity within individual samples and between different samples, respectively. Alpha diversity, assessed using the species richness estimator (Chao1) and diversity indices (Shannon and Simpson), was visualised for both 5-aza-treated and untreated (BOD) samples through box plots, each representing the diversity distribution within a population (Figure S4a–c). The species richness values (Chao1) lay between 232 and 780 across samples, indicating remarkable variation, viz., the number of bacterial taxa detected between 5-aza-treated and untreated samples. Fewer bacterial taxa were found in BPH after 5-aza treatment (mean Chao1 index value was 582 and 359 for BOD (untreated) and 5-aza-treated insects, respectively). This was also supported by the alpha rarefaction curve analysis and diversity estimates based on observed ASVs. Here, fewer ASVs were observed for 5-aza-treated insects (Table S5). In addition, the Shannon and Simpson diversity index values also differed significantly between 5-aza-treated and untreated samples (Table S5, Figure S4b,c). The Shannon diversity index ranged from 3.86 to 1.627, while the Simpson diversity index varied between 0.832 and 0.276 (Table S5), reflecting variations in taxa abundance (evenness) among BPH populations across samples (Figure S4b,c).

The beta diversity analysis indicated differences in bacterial diversity and composition between samples. This was evident from the 3D principal coordinate analysis plots, where each axis represents variation between samples. Here, the X-axis (PC1) depicted the highest degree of variation (87.45%), followed by the Y-axis (PC2; 8.279%) and the Z-axis (PC3; 2.169%) (Figure S4d–g). Additionally, the significance of the clustering pattern was confirmed by Adonis’s test (*p*-value < 0.05) based on weighted and unweighted unifrac distances (Table S6).

Further, MetaStat analysis identified >15 bacterial taxa, which differed significantly between the two groups (i.e., 5-aza-treated and untreated) at the adjusted *p*-value cut-off of ≤0.05 (Table S7, Figures S5 and S6). Further, LEfSe analysis revealed taxa with a significant effect size (with LDA score > 4; Figures S5 and S6). It was observed that, while the gut microflora of BOD (untreated) insects was predominated by *Ochrobactrum*, *Methylophilus*, *Elizabethkingia*, *Helicobacter*, and *Pseudonocardia*, 5-aza-treated insects had an abundance of *Brevibacterium*, *Achromobacter*, *Bacillus*, *Reyranella*, *Delftia*, *Rhizobium*, *Stenotrophomonas*, and *Acinetobacter*.

**Figure 5:**
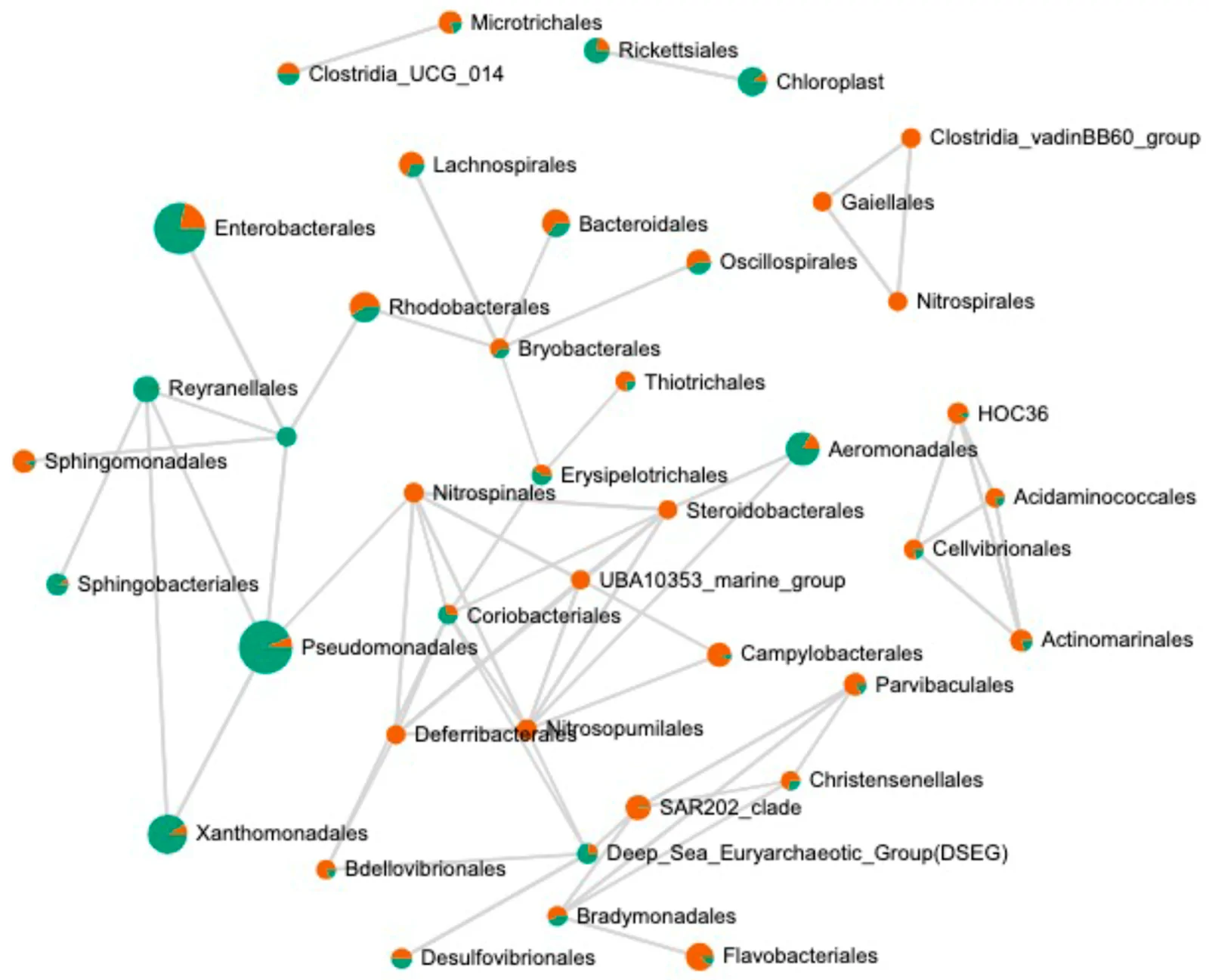
Correlation network analysis of microbiota present in 5-aza-treated (Aza) and untreated (BOD) insects using the SparCC algorithm. Networks between abundant sequences at the order level built from SparCC correlation coefficients (*p*-value cut-off < 0.05) are indicated. While the nodes represent bacterial taxa, the edges indicate the correlation between them. The pie chart depicted within nodes represents the relative proportion of the bacterial taxa in 5-aza-treated (green) and BOD (control; orange) groups, and the circle size represents the read number/abundance.

**Figure 6:**
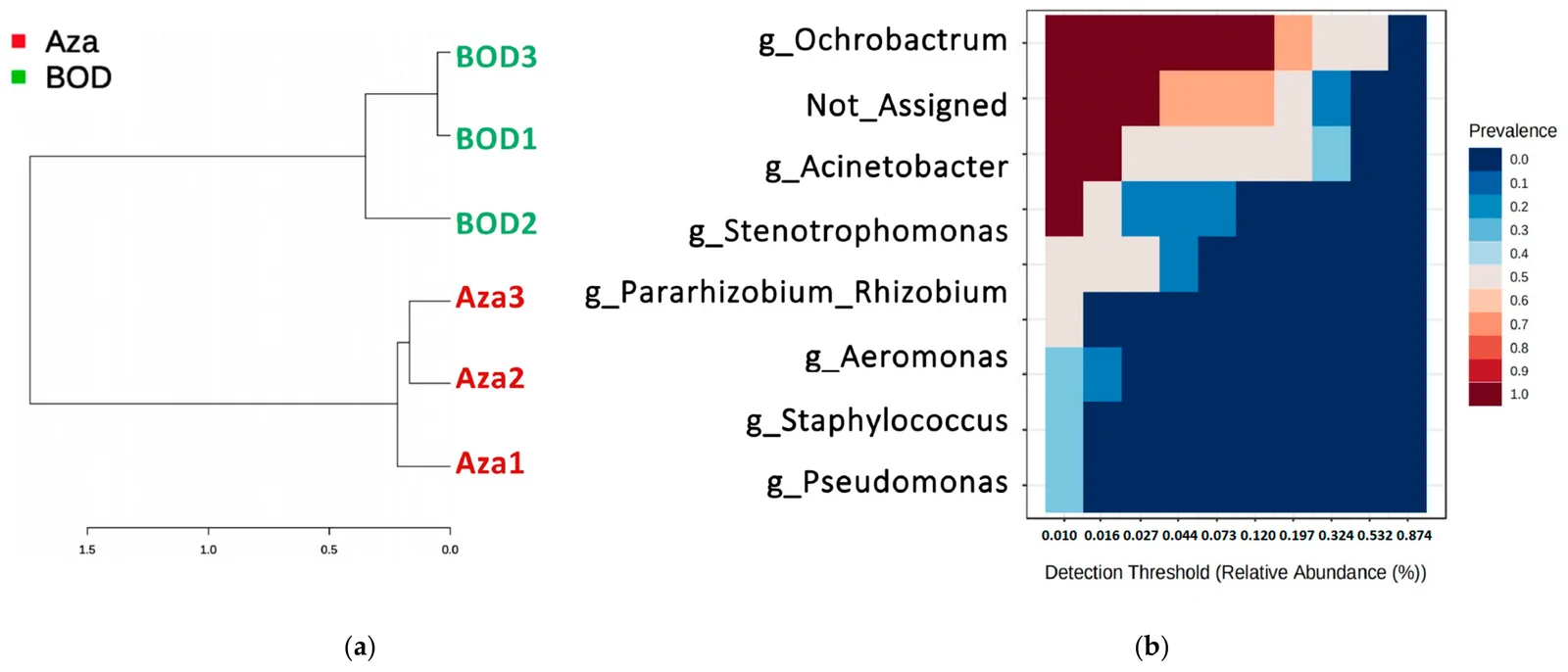
Phylogenetic relatedness of aza-treated and BOD samples based on microbiome constituents and the elements of the core microbiome. (**a**) Dendrogram showing hierarchical relationship between samples (calculated using the Ward’s algorithm), based on bacterial taxa composition and abundance. Scale indicates Euclidean or dissimilarity distances. (**b**) Constituents of the core microbiome representing the bacterial taxa characteristic of 5-aza-treated (Aza) and untreated (BOD) insects. Heat map indicates the relative prevalence of bacterial taxa across treated and untreated samples. The X-axis represents the detection thresholds (indicated as relative abundance) from lower (left) to higher (right) abundance values. Colour shading indicates the prevalence of each bacterial taxon among samples for each abundance threshold. With increase in the detection threshold, the prevalence decreases.

The comparison of microbial community structure and their interactions also revealed a significant decline in the overall microbial diversity following 5-aza treatment (Figure 5), confirming and corroborating the results obtained from alpha diversity estimates. Furthermore, based on microbiome composition, diversity, and relative abundance of bacterial genera within samples, the hierarchical clustering analysis grouped 5-aza-treated and untreated samples into two discrete clusters, differentiating the two groups (Figure 6a). In addition, core microbiome analysis revealed bacterial taxa that were retained even upon exposure to 5-aza. Some of the bacterial taxa identified were *Ochrobactrum*, *Enterobacter*, *Pseudomonas*, *Rhizobium*, *Acinetobacter*, *Stenotrophomonas*, and *Staphylococcus* (Figure 6b).

#### 2.2.4. Differential Metabolic Capacities of the Bacterial Community Present in 5-Aza-Treated Insects

Considering the remarkable changes observed, viz., microbiome structure and composition upon 5-aza treatment, we deemed it pertinent to delineate the cause-and-effect relationship with the insect host. Moreover, elucidating the metabolic networks can be used to model and compare the actual biochemical processes carried out by the different microbes present in the two samples. In this regard, gut microbial functional profiles predicted based on *16S rRNA* metagenomics data differed significantly between 5-aza-treated and control (BOD) insects (Table S8). A total of 669 KEGG IDs (with each ID corresponding to a metabolic process) were found to be influenced by 5-aza treatment when compared with the control (untreated) samples (*p*-value < 0.05; Figure S7). However, the functional profiling of microbial communities at level 1 KO categories suggested that, in both 5-aza-treated and untreated samples, >50% of bacteria were involved in “metabolism”, followed by “environmental information processing” (i.e., >20%), and “genetic information processing” (i.e., >15%; Figure S8a). Interestingly, although the overall contribution of bacteria towards these processes remained unchanged (at level 1) between 5-aza-treated and untreated (BOD) samples, the titres of bacterial taxa contributing to each process decreased after 5-aza treatment (Figure S8b).

**Figure 7:**
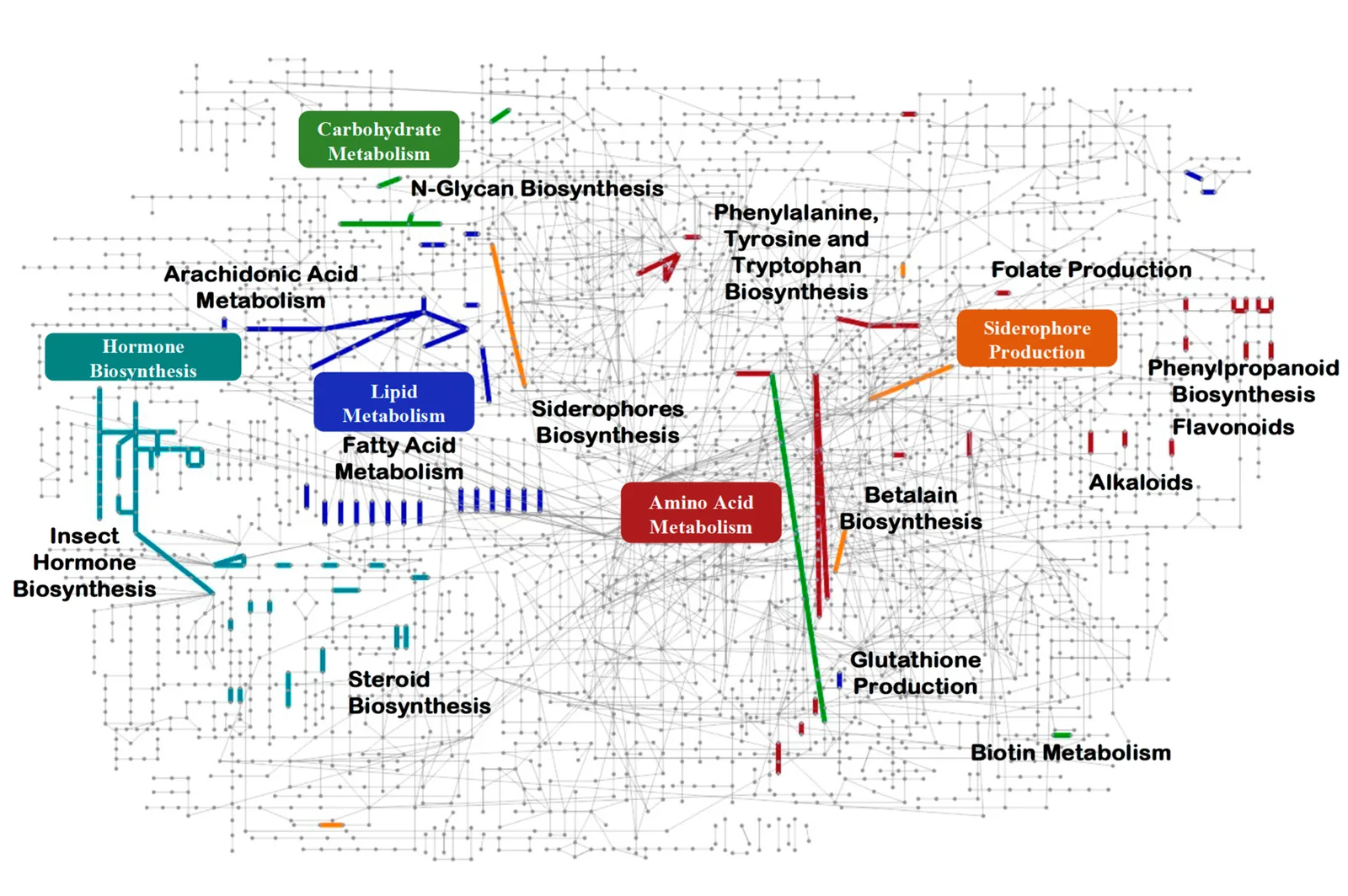
Network analysis of the microbiome present in the aza-treated and BOD samples. Predicted KEGG global metabolic network with differentially regulated pathways (major categories are highlighted by coloured boxes) present in 5-aza-treated (Aza) and untreated (BOD) samples. Sub-pathways within each major category are highlighted and the colour indicates the respective categories they belong to. Black edges represent common pathways that remained unchanged between the two groups Aza and BOD. Red and blue lines show positive and negative correlations, respectively. Yellow, pink and blue nodes represent pathways in decreasing order of their significance.

Usually, bacterial taxa constituting the microbiome act as communities and are considered drivers of the complex metabolic interactions that exist between the host and these microbial partners. Therefore, based on KEGG metagenome predictions and the relative titres of bacteria contributing to these processes, the functional pathway reconstruction analysis was conducted for both 5-aza-treated and untreated samples. Results predicted >85 differentially regulated pathways in 5-aza-treated and untreated samples (at *p*-value cut-off < 0.05; Table S9 and Figure 7) based on *16S rRNA* data. It was observed that 5-aza-induced alterations in microbiome composition largely influenced the amino acid synthesis, carbohydrate metabolism (TCA cycle), respiration (Glycolysis), lipid metabolism, vitamin B (biotin) biosynthesis, and oxidative metabolism in BPH (Figure 7).

Furthermore, as underlying mechanisms controlling biological functions and expression of various phenotypes usually involve a large group of metabolites and/or pathways, the partial correlation networks were built using the novel debiased sparse partial correlation (DSPC) algorithm. Based on identified metabolic features, the DSPC analysis generated four subnetworks having high biological relevance (i.e., with correlation cut of value < 0.05, degree value > 1, betweenness > 50) and connectivity patterns visualised as weighted networks. Of these, subnetwork1 consisted of 58 nodes (representing the metabolic features) and 116 edges (depicting the nature of correlations between them) (Figure S9a). Some of the predicted cellular and metabolic processes that were crucial for the flow of information within the network were signal transduction mechanisms, sulphur metabolism, biotin metabolism, folate biosynthesis, drug metabolism, phosphatidylinositol signalling system, arachidonic acid metabolism, lipid metabolism, etc. (Table S10 and Figure S9b).

#### 2.2.5. 5-Aza Treatment Impacted BPH’s Innate Immune System

**Figure 8:**
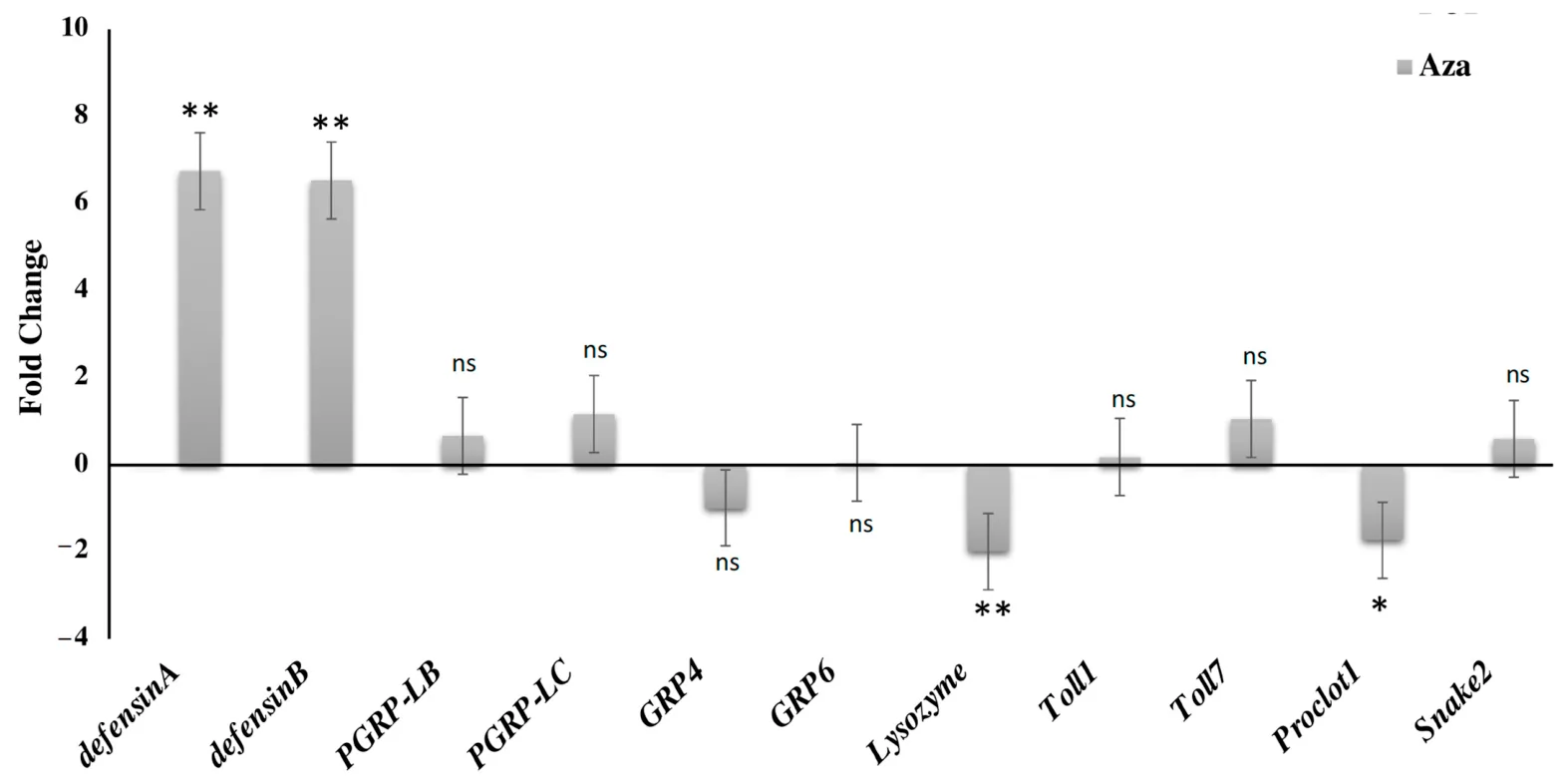
Quantitative RT-PCR-based analysis of the expression of immune-related genes in BPH after treatment with 5-azacytidine. BOD population (untreated) was used as control. The relative gene expression was normalised to the expression level of *actin*. The results were analysed using the 2^−ΔΔCt^ method and the expression level is displayed as relative expression values based on the relative standard curve method. The analysis is based on three biological and three technical replicates. Relative fold change values of Aza samples were obtained using the expression in BOD samples as the baseline. Genes showing significant change in their expression profiles are marked (**): highly significant, >2-fold change; (*): significant, 2-fold change; (ns): not significant, <2-fold change.

Following treatment with 5-aza, remarkable changes in the mRNA levels in BPH were observed (Figure 8). Post 5-aza treatment, while the expression levels of *defensinA* and *defensinB* increased by >6-fold, *Toll7* showed slight upregulation (>1.5-fold increase). However, in contrast, *GRP4*, *Proclot1*, and *Lysozyme* showed significant downregulation in their expression levels (i.e., >2-fold decrease) in 5-aza-treated insects. In addition, *PGRP*-LB, *PGRP*-LC, *GRP6*, *Toll1*, and *Snake2* showed no significant change in their mRNA levels upon exposure to 5-aza.

#### 2.2.6. 5-Aza and Antibiotic Treatment Impacted Lipid Metabolism in BPH

**Figure 9:**
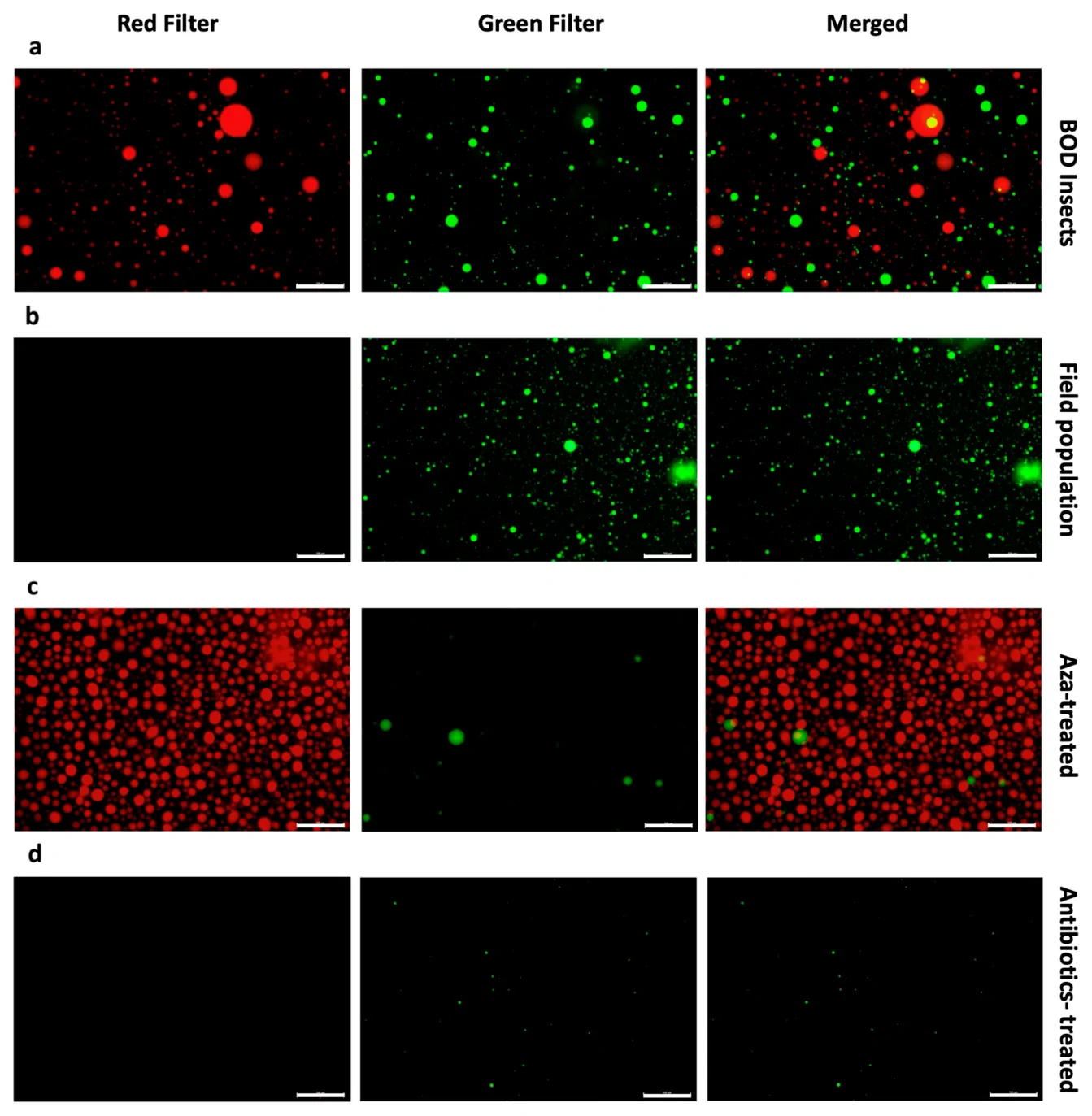
Nile Red staining of the lipid storage droplets in the fat body of BPH adults. Dissected fat bodies of BPH insects, (**a**) BOD control, (**b**) insects collected from rice field, (**c**) aza-treated and (**d**) antibiotic-treated were stained with Nile Red for 1 h and observed using a Nikon Eclipse Ci fluorescence microscope using green (488 nm) and red (543 nm) filters. Images were acquired at both wavelengths and captured using a Nikon digital camera DS-Ri2. Neutral lipids stain green and polar lipids stain red. Magnification bar = 100 µm. The analysis was carried out in triplicate.

Fat bodies in 5-aza-treated insects accumulated a more prominent and higher density of lipid droplets than those observed in the fat bodies of the control individuals. In contrast, results indicated that antibiotic treatment disrupted the fat metabolism in BPH individuals, which led to an overall decrease in the lipid content (Figure 9). Additionally, while both wild- and lab-reared BPH individuals demonstrated a rich lipid profile, the lab-reared (inbred) insects contained a higher percentage of polar lipids (characterised by red fluorescing droplets), whereas the wild population had more elevated amounts of neutral lipids (indicated by green fluorescing droplets; Figure 9).

## 3. Discussion

This study provides experimental evidence that perturbation of either the gut microbiome or host DNA methylation is associated with reciprocal changes in the other system, supporting a relationship between the microbiome and epigenome in the brown planthopper. While similar interactions have been reported in vertebrates [22,32,33,34,35], experimental evidence of comparable interactions in insects remains limited. Our findings help address this knowledge gap and provide a foundation for future mechanistic studies of microbiome–epigenome interactions in insects.

Antibiotic-mediated gut dysbiosis altered global and gene-specific DNA methylation patterns (Figure 1), suggesting that gut microbiota contribute to host epigenetic homeostasis. These changes may result from altered microbial metabolites, disruption of host metabolic processes, or antibiotic-induced physiological stress, all of which are implicated in epigenetic regulation [2,3,10,14,15,16,36]. Consistent with this, antibiotic-treated insects showed depleted lipid reserves (Figure 9), suggesting altered metabolic homeostasis that could indirectly influence DNA methylation. However, stress itself can also modify the epigenetic status of BPH [1]; therefore, it is possible that observed changes arose from antibiotic-induced stress independent of microbiome depletion. To confirm, future studies employing microbiome recolonisation or targeted microbial manipulation will be required.

Altered methylation of several stress-responsive genes further suggests that the gut microbiome may influence pathways involved in stress adaptation. Similar associations between microbial disruptions and host genome methylation have been reported in other organisms [24,26,37,38,39,40,41], supporting the possibility that microbial communities may shape host epigenetic landscapes. However, because gene expression and promoter methylation were not examined, these findings require additional work integrating genome-wide methylation profiling, transcriptomics, and functional validation to determine how microbiome-induced methylation changes influence stress adaptation in insects.

Further evidence for bidirectional microbiome–epigenome interactions was obtained by perturbing host DNA methylation using 5-azacytidine and examining its effects on the gut microbiome. DNA methylation disruption altered microbial community composition, increasing bacterial abundance while reducing microbial diversity. This suggests that host epigenetic regulation maintains microbial homeostasis, and disruption of methylation creates conditions that favour the expansion of opportunistic bacteria. Typically, microbial titres are regulated by insect innate immunity [42,43], which is under epigenetic control [10,44,45,46]. Thus, it is plausible that the azacytidine-induced changes in the methylation status of immune-related genes impacted their activity, thereby enabling the opportunistic bacteria to proliferate. To confirm, we examined the activity of immune-related genes in BPH after exposure to 5-azacytidine.

DNA methylation perturbation altered the expression of a subset of immune-related genes involved in bacterial recognition and clearance [47], indicating that epigenetic processes contribute to maintaining gut microbial balance. Conversely, the marked induction of antibacterial peptide genes (*defensinA* and *defensinB*) likely represents a secondary immune response to increased bacterial proliferation (i.e., an attempt to restore gut homeostasis following an abrupt rise in pathogenic bacteria following 5-aza treatment). Functional studies, including promoter methylation analyses and gene-specific validation, will be required to establish the mechanisms linking DNA methylation to immune regulation.

Interestingly, while the microbial titres rose sharply following 5-azacytidine treatment, overall bacterial diversity declined, indicating gut community restructuring rather than uniform bacterial proliferation. This shift reflects altered host physiology, that selectively favours bacteria capable of exploiting the modified gut environment, as well as disruptions in interactions among resident microbial taxa. Moreover, this microbial reorganisation may also compensate for lost or disrupted host metabolic pathways following 5-aza treatment.

In this regard, we examined bacterial functions and contributions in 5-aza-treated and untreated insects towards host survival. Under untreated BPH, the gut is dominated by beneficial microbes such as *Ochrobactrum* ([48]; xenobiotic detoxification), *Methylophilus* ([49]; mitigates stress), *Pseudonocardia* ([50]; host protection), *Helicobacter* ([51]; maintenance of symbiosis), and several members of Rhizobiales ([52]; nitrogen utilisation and facilitates feeding). In contrast, 5-aza-exposure increased pathogenic taxa associated with folate biosynthesis (e.g., *Brevibacterium*; [53]), degradation of organophosphorus insecticides (e.g., *Pseudomonas*), and Chlorpyrifos resistance (e.g., *Delftia*; [54]). This microbiome restructuring following methylome perturbation may represent an adaptive response that partially compensates for altered host physiology.

Functional profiling of bacterial communities in BPH predicted pathways associated with lipid metabolism, cysteine and methionine metabolism, sulphur metabolism, drug metabolism, folate biosynthesis, biotin metabolism, and signal transduction in BPH that were differentially regulated in 5-aza-treated and untreated insects. Nile Red assays strengthened these predictions. As predicted, 5-aza treatment significantly altered lipid metabolism. Lipids are central to insect energy homeostasis and also play important roles in immune function by providing energy for cellular defence responses and serving as precursors for immune-related molecules [55,56,57,58,59,60,61]. Therefore, the increased lipid content observed in 5-aza-treated insects may represent a physiological response to the proliferation of pathogenic bacteria or to metabolic disturbances associated with altered DNA methylation. Together, these findings suggest that microbiome–epigenome interactions extend beyond immune regulation to influence host metabolism.

Interestingly, both total lipid content and lipid types differed across treatments and populations. While insects reared under controlled (favourable) conditions have lower energy demands that favour lipid storage, field-collected insects face hostile environments, requiring higher energy and the mobilisation of lipid reserves. This rationale likely explains the differing triglyceride (neutral lipid) levels observed across samples. Although these observations were not the primary focus of this study, they further highlight the dynamic relationship between environmental conditions, host metabolism, and physiological state.

Several predicted microbial functions further suggest potential compensatory interactions between the gut microbiome and the host following 5-azacytidine treatment. In particular, changes in the folate biosynthesis pathway may be linked to an increase in *Bifidobacterium* titre upon 5-aza exposure. Studies have shown that *Bifidobacterium* species can potentially influence the folate biogenesis pathway which supplies the methyl groups required for DNA, RNA, or protein methylation [5,53,62,63,64]. If so, this suggests that bacterial symbionts likely compensate for 5-aza-induced perturbations in BPH. Likewise, predicted alterations in sulphur metabolism may have significantly affected the abundance of microbes such as *Methylophilus* [65], thereby explaining the marked decline in its titre following 5-aza treatment. And alterations in drug metabolism pathways may explain the shifts observed in bacterial taxa such as *Pseudomonas* and *Delftia*, which are implicated in biodegradation. Collectively, these observations suggest that microbial communities may partially compensate for host metabolic perturbations following methylome disruption [66]. However, because these functional inferences are based on 16S rRNA-derived predictions, they require validation through integrative metagenomic, transcriptomic, proteomic, and metabolomic analyses. In addition, the pharmacological effect of 5-aza treatment, beyond epigenome perturbation, needs to be evaluated to obtain conclusive results.

Collectively, our results provide experimental evidence supporting a bidirectional association between the gut microbiome and host epigenome in insects, thereby addressing an important knowledge gap in the field. By independently perturbing the gut microbiome and host DNA methylation, we demonstrate that changes in one component are consistently associated with alterations in the other, supporting the existence of bidirectional microbiome–epigenome interaction. Perturbation of the gut microbiome altered host DNA methylation, while disruption of host DNA methylation reshaped microbial community composition, immune gene expression, and predicted microbial metabolic functions. Although these findings support reciprocal microbiome–epigenome interactions, the molecular mechanisms underlying these associations remain unresolved. In particular, the microbial metabolites that influence host epigenetic regulation, the host signalling pathways involved, and the extent to which these interactions directly contribute to stress adaptation require further investigation. Moreover, because both antibiotics and 5-azacytidine can exert physiological effects independent of microbiome depletion or DNA methylation, future studies employing targeted microbiome manipulation, microbiome recolonisation, genome-wide methylation profiling, functional genomics, metabolite supplementation, and multi-omics approaches will be essential to establish causal mechanisms. Such studies will provide a more comprehensive understanding of how microbiome–epigenome interplay contributes to insect adaptation and may ultimately facilitate the development of novel strategies for sustainable insect pest management.

## 4. Materials and Methods

### 4.1. BPH Populations

A population of BPH (*Nilaparvata lugens*; referred to as the BOD population), which was established from two males and two females collected from rice fields in Delhi, was reared on a susceptible rice variety TN1 (Taichung Native 1) in a BOD incubator at ICGEB, New Delhi. The incubator was maintained at 28 °C with a photoperiod of 16 h light and 8 h dark for over 50 generations. This population served both as the control group and as the source from which insects subjected to antibiotic and azacytidine treatments were derived.

### 4.2. Antibiotic Treatment

To obtain insects with no/minimal bacterial symbionts, adult BPH (~10 insects/tube) were maintained on an artificial diet (for details refer to Fu et al. [67]) supplemented with an antibiotic cocktail for 48 h. In total, 50 mL of the artificial diet contained four antibacterial agents, namely, 0.01 g Neomycin sulfate (MP Biomedicals, Santa Ana, CA, USA), 0.05 g Aureomycin (BioServ, Flemington, NJ, USA), 0.003 g Streptomycin (Sigma), and 0.0025 g Rifampicin (Sigma, Philippines). The insects were reared in glass cylinders (open at both ends; 3 cm length and 2 cm diameter) sealed at both ends by a double layer of Parafilm. The artificial diet was placed between the two layers of Parafilm. The media (artificial diet with antibiotic cocktail) were replenished every 12 h. In addition, tubes containing only standard media (without antibiotics) were also set up and used as controls. Insects surviving after 48 h were collected from both the control and antibiotic-treated tubes and are referred to as STD and ENDO, respectively, and stored in absolute ethanol at −20 °C until further use.

### 4.3. Genomic DNA Isolation from Antibiotic-Treated Insects

Genomic DNA was extracted from BPH individuals using the GF-1 tissue DNA extraction kit (Vivantis, Subang Jaya, Malaysia) in accordance with manufacturer’s instructions. DNA concentration was measured with a NanoDrop Spectrophotometer (Thermo Fisher Scientific, Waltham, MA, USA) and quality was assessed by electrophoresis on a 0.8% TBE agarose gel [68].

### 4.4. Quantitation of the Microbial Symbionts in Antibiotic-Fed and Control BPH Using Semi-Quantitative PCR

To quantify the amount of bacterial load in BPH individuals post-antibiotic treatment, a semi-quantitative PCR assay specifically targeting the V3–V4 region of *16S rRNA* was designed. This assay was based on the premise that insects with minimal microflora would contain fewer copies of the target sequence, which can be measured by semi-quantitative PCR and when compared to the insects (untreated) with intact microbiome, would enable the estimation of percentage reduction in bacterial titres. In this regard, the most promising primer pair (V3-341F 5′-CCTACGGGNGGCWGCAG-3′ and V4-805R 5′-GACTACHVGGGTATCTAATCC-3′) for microbiome profiling, as suggested by Klindworth et al. [69], was used for amplification. PCR amplification reactions were set up for both the antibiotic-treated and control insects. The amplification obtained from control insects served as a comparator to estimate the antibiotic-mediated reduction in bacterial load in BPH. In addition, to account for variations arising due to the amounts of input template DNA, an independent reference (internal control) was established for the BPH actin gene (Accession: KU196668.1). The primer pair used for *actin* was ACT-mod F 5′-TGCGTGACATCAAGGAGAAGCTG-3′ and ACT-mod R 5′-GTACCACCGGACAGGACAGT-3′. The amplification profile used for both *16S rRNA* and *actin* was as follows: an initial denaturation of 2 min at 94 °C followed by 25 cycles (PCR amplifications were performed using 20, 25, 30, and 35 cycles to determine the endpoint of amplification. And based on the results, 25 cycles were found to remain within the linear amplification range) of 30 s at 95 °C, 30 s at 58 °C, and 45 s at 72 °C, with a final extension of 2 min at 72 °C (Gupta et al. [30]). The PCR conditions were the same for both the *16S rRNA* and *actin*. The PCR products were run on a 1% agarose gel and visualised using a gel documentation system (Alpha Imager, Cell Biosciences, Cambridge, UK). Quantification of the PCR product was done based on the intensity of the band (analysed using the ImageLab software 6.0.2) obtained for the *16S rRNA* fragment, while using the intensity of the amplified actin gene product from each sample for normalisation.

### 4.5. Estimation of the Impact of Antibiotic-Mediated Disruption of the Microbiome on the BPH Methylome

#### 4.5.1. Global DNA Methylation Analysis of BPH Exposed to Antibiotics

The effect of microbiome disruption on host DNA methylation was assessed through global DNA methylation analysis using the MethylFlash Global DNA Methylation ELISA Easy Kit (Epigentek, Farmingdale, NY, USA). Post-antibiotic treatment, genomic 5-mC levels in BPH were quantified and compared with those of untreated (control) insects, employing three biological and three technical replicates for analysis. Each biological replicate contained genomic DNA from 8 individuals (for both control and treated insects; 2 macropterous males, 2 macropterous females, 2 brachypterous males, and 2 brachypterous females) combined in equal proportions.

#### 4.5.2. Analysing the Methylation Patterns of Stress-Responsive Genes in BPH

Alteration in DNA methylation patterns upon antibiotic exposure was estimated at single-nucleotide resolution for a few stress-responsive genes in the BPH genome. Genes analysed in this study were shortlisted based on their relevance and functional utility for BPH (for details, refer Table S1 Gupta and Nair [27]). Further, it has been reported that the activity of these genes is epigenetically controlled [27], and hence they were chosen for studying the impact of gut dysbiosis on epigenetic profile of the insect host. Additionally, since insect DNA methylation primarily occurs in the CpG islands, we limited our analysis to these regions. The CpG islands within the selected stress-responsive genes were identified using EMBOSS CpGplot software (Version 6.6.0) Subsequently, targeted bisulfite sequencing was employed to screen these islands for methylation marks induced by antibiotic treatment.

##### Bisulfite Treatment of BPH DNA

For the bisulfite conversion, genomic DNA from 8 individuals (for both control and treated insects) was combined in equal proportions. Each pool included DNA from 2 macropterous males, 2 macropterous females, 2 brachypterous males, and 2 brachypterous females. Bisulfite conversion was performed using the EpiJET Bisulfite Conversion Kit (Thermo Fisher Scientific, Waltham, MA, USA) with 200 ng of pooled DNA per reaction. Following bisulfite treatment, DNA recovery was quantified using a Qubit 4.0 fluorometer (Invitrogen, Carlsbad, CA, USA), and conversion efficiency was evaluated based on the conversion rate of a control reaction.

The control reaction produced a 283 bp PCR amplicon corresponding to the actin gene of BPH, amplified using ACT-mod F and ACT-mod R primers. The resulting PCR product was resolved on a 1% agarose gel and subsequently purified with a nucleic acid extraction kit (Vivantis GF-1, Subang Jaya, Malaysia), and subsequently subjected to bisulfite treatment. As the amplified fragment is unmethylated, all cytosine residues are expected to convert to uracil post-bisulfite treatment. The PCR product was processed in parallel with the test samples.

Following bisulfite treatment, the 283 bp amplicon was again PCR amplified using the same primer pair on the bisulfite-treated *actin* fragment template. The resulting product was cloned into the pCR4-TOPO vector using the TOPO TA Cloning Kit (Invitrogen, Carlsbad, CA, USA). Plasmid DNA from positive clones was then extracted and sent to M/s Macrogen Inc. (Seoul, Republic of Korea) for sequencing. The chromatograms were analysed using MacVector (version 15.5) and compared with the original *actin* sequence. The efficiency of bisulfite conversion was evaluated based on the sequencing data as the ratio of converted to non-converted cytosines.

##### Bisulfite PCR Amplification, Gel Purification and Quantification of PCR Products

The PCR primers targeting bisulfite-converted DNA for each gene were designed from the regions adjacent to their respective CpG islands (for details, refer to Table S7 Gupta and Nair [29]). The CpG islands associated with the chosen stress-responsive genes were amplified from both control and antibiotic-treated samples, using 15 ng of bisulfite-treated DNA as the template.

The PCR conditions varied among the genes and were as follows: initial denaturation at 95 °C for 2 min, followed by 30 cycles of 30 s at 95 °C, 1 min at 45–62 °C, 1 min at 70–72 °C, and a final elongation at 72 °C for 2 min. Each PCR reaction contained bisulfite-treated DNA template (15 ng), dNTPs (200 μM), Taq DNA polymerase (0.6 U), 1× buffer, and primers (13 μM). The PCR products were run on a 1% agarose gel, purified and then quantified on a Qubit 4.0 fluorometer (Invitrogen, Carlsbad, CA, USA). Subsequently, PCR fragments representing various genes under treatment were equally pooled and forwarded to M/s IMGM Laboratories GmbH, Bavaria, Germany, for library preparation and Illumina-MiSeq 250 paired-end sequencing.

##### Raw Data Processing and Quality Check

Following sequencing, reads were filtered, an initial quality check (QC) was performed, 3′-end adapters were trimmed, and the reads were demultiplexed, using the indices and corresponding sample IDs. The resulting R1 and R2 files (in the fastq format) were subjected to downstream analyses.

##### Data Analyses

Post-quality-based filtering and adapter trimming, the R1 and R2 reads were merged and subsequently aligned to the reference sequence (i.e., DNA sequence corresponding to each gene). The alignment files thus generated were sorted, demultiplexed and indexed, and mapping statistics were estimated. Thereafter, alignment and methylation calling were performed to assess the overall methylation status and coverage using Bismark (v0.20.1). After alignment, the methylation data for each cytosine examined across all three contexts were extracted. Subsequently, the statistical analysis was performed on the percentage of methylation across the chosen genes in all three contexts.

Further, we did the differential methylation analysis and computation of relative methylation scores for each site across samples, and the resulting output files were used to create line graphs for visualisation and interpretation. Coverage files, containing methylation counts and coverage for individual cytosines, were generated and subjected to statistical analyses. Variations in methylation levels between antibiotic-treated and control populations were assessed via Fisher’s exact test. A sliding-window smoothing approach was used to adjust the data to account for sequencing depth and biological variation, thereby making differential methylation calling robust against low-coverage sites. Here, the *p*-value was calculated based on the chi-square test, which, based on the sliding linear model, was subsequently adjusted to a *q*-value. Post-*q*-value estimation, differentially methylated cytosines showing a difference of ≥25% and a *q*-value ≤ 0.01 were detected.

### 4.6. Azacytidine Treatment

Our earlier study showed that >40 μM 5-aza is lethal for BPH [27]. Therefore, to investigate whether and how the disruption of BPH methylome impacts its microbiome, BPH individuals were treated with 40 μM azacytidine. Adult BPH (~10 insects from the BOD population per tube) were fed for 48 h on an artificial diet supplemented with 5-azacytidine (40 μM; Sigma-Aldrich, St. Louis, MO, USA) and phenol red (0.2 μg/μL) as a feeding indicator. The artificial diet was replenished every 12 h. For this, the insects were transferred to fresh tubes containing artificial diet, 5-azacytidine and phenol red (Gupta and Nair [27]). Live insects after azacytidine treatment were collected, preserved in absolute ethanol at −20 °C for downstream processing.

### 4.7. Quantification of DNA Methyltransferase (DNMT) Enzymatic Activity

The efficacy of 5-azacytidine treatment was evaluated by measuring the DNMT activity in the treated insects. In this regard, the nuclear protein was extracted using an EpiQuik Nuclear Extraction Kit (Epigentek, Farmingdale, NY, USA) and quantified using the Bradford protein assay kit (Bio-Rad, Hercules, CA, USA) as per the manufacturer’s instructions. DNMT activity was measured with 3 μg of the nuclear protein extract from each sample (i.e., treated and untreated) using the EpiQuik DNA Methyltransferase Activity/Inhibition Assay Kit (Epigentek, Farmingdale, NY, USA) following the manufacturer’s protocol. Activity levels in the treated insects were compared with those of controls, and the percentage reduction in DNMT was calculated. The experiment was conducted in triplicate with 10 insects per replicate.

### 4.8. Isolation of Genomic DNA from 5-Aza-Treated Insects

Total genomic DNA was extracted from individual adult insects (N = 48) from 5-aza-treated and control groups following the same protocol as previously mentioned.

### 4.9. Quantification of Methylation Levels

To verify the effect of 5-aza treatment on the methylation status of the loci under investigation, we utilised a modified methylation-sensitive restriction assay, followed by CpG island amplification-representational difference analysis [70], which involved digesting genomic DNA from both treated and control samples using methylation-sensitive/insensitive isoschizomeric restriction enzyme pairs, followed by semi-quantitative PCR. This assay is based on the premise that only genes with homogeneously methylated alleles remain unrestricted when a methylation-sensitive restriction enzyme is used and, hence, can be amplified and quantified by PCR. In contrast, the partly methylated and unmethylated alleles would be digested, disrupting the target sequence and leading to a reduction in copy number, which can be quantified by semi-quantitative PCR.

Each restriction reaction included pooled genomic DNA (200 ng), buffer (1×), restriction enzyme (2 U), and sterile water (final volume 25 μL), and was incubated for 6 h at 37 °C for HpaII, MspI, and AatII; 55 °C for BsmAI and FatI; 50 °C for BstCI. Subsequently, semi-quantitative PCR was conducted using gene-specific primers listed in Table S1. For each sample, the PCR amplification was carried out for both digested DNA samples (i.e., one treated with a methylation-sensitive and the other with a methylation-insensitive restriction enzyme). An undigested control (UC) was also included to normalise for differences in template DNA quantity across PCR reactions and to determine the initial abundance of the target sequence. All PCR reactions (final volume 20 μL) consisted of 200 μM dNTPs, 0.6 U Taq DNA polymerase (Bangalore Genei, (India) Pvt. Ltd., Bengaluru, India), 1× Taq buffer, and 13 μM each of forward and reverse primers. PCR was performed using 20 ng of the genomic DNA as a template in both digested and undigested reactions. The PCR amplification profile was: initial denaturation at 95 °C for 5 min, followed by 25 cycles of denaturation at 95 °C for 30 s, annealing at 55–60 °C for 30 s, extension at 72 °C for 30 s and a final extension of 72 °C for 2 min. The PCR products were run on a 1% agarose gel. The relative intensity for each PCR amplified fragment in digested (methylation-sensitive/insensitive restriction digestion) and undigested (control) lanes obtained for each gene was quantified using Image Lab software (v6.0.1; Bio-Rad Laboratories, Hercules, CA, USA). The PCR fragment’s intensity from the undigested control (UC), unaffected by restriction digestion and thus reflecting 100% methylation, served as the reference point. Consequently, the methylation level of each sample was determined by normalising its band intensity to that of the corresponding PCR fragment amplified from the UC template.

### 4.10. Quantification of Bacterial Load Following Azacytidine Treatment

To quantify the amount of bacterial load in BPH individuals (N = 48; 2 technical replicates, adults) following azacytidine treatment, a semi-quantitative PCR assay was performed using the V3-341F and V4-805R primer pair (details mentioned above). PCR was performed using genomic DNA obtained from BPH individuals treated with azacytidine and controls (untreated) for 25 cycles (lesser number of cycles used to ensure that measurements of generated PCR products were made at the linear phase of the PCR amplifications). The amplification obtained from the control insects served as a comparator to measure the alteration(s) in bacterial titres in BPH after azacytidine treatment. The assay protocol was the same as mentioned before. Student’s *t*-test (*p* ≤ 0.05) was performed to determine if the difference in bacterial titre between the two groups (i.e., 5-aza-treated and control insects) was significant.

### 4.11. Estimation of the Impact of 5-Aza-Induced Alterations in the BPH Methylome on Its Microbiome Structure and Composition

*16S rRNA* Amplicon Metagenomic Sequencing was carried out to identify differentially present bacterial taxa and their relative abundance in 5-aza-treated and control insects. In this regard, genomic DNA (from 5-aza-treated and control insects) from eight BPH individuals/replicate was pooled in equal amounts and sent to M/s Novogene, Singapore, for PCR amplification, library preparation, and sequencing. The analysis was carried out in triplicate.

#### 4.11.1. Construction of *16S rRNA* Library and NovaSeq Sequencing

The PCR amplification of the targeted *16S rRNA* (V3-V4) region was carried out using specific primers (V3-341F 5′-CCTAYGGGRBGCASCAG-3′ and V4-806R 5′-GGACTACNNGGGTATCTAAT-3′) with barcodes, and the PCR products of appropriate size were selected by 2% agarose gel electrophoresis. For library preparation, an equal amount of PCR products from each sample was pooled, end-repaired, A-tailed and further ligated with Illumina adapters. The library was quantified using a Qubit fluorometer (Invitrogen, Carlsbad, CA, USA) and real-time PCR, and size distribution analysis was carried out on a bioanalyzer (Agilent, Santa Clara, CA, USA). Libraries were sequenced on the Illumina NovaSeq sequencing platform to generate 250 bp paired-end raw reads.

#### 4.11.2. Demultiplexing, QC Filtering, and Generation of Effective Tags

Paired-end reads obtained after sequencing were subjected to demultiplexing, adapter trimming, QC filtering, and noise reduction. Next, the paired-end reads were merged using FLASH (Version 1.2.11, http://ccb.jhu.edu/software/FLASH/; accessed on 15 July 2023). This was followed by quality filtering to obtain high-quality Clean Tags. The Clean Tags were then mapped to the reference database (Silva) for detection of the chimaera sequences, which were filtered out to generate the Effective Tags.

#### 4.11.3. Amplicon Sequence Variants (ASVs) Analysis—Denoise, Annotation, and Phylogenetic Relationship Reconstruction

To obtain initial ASVs, denoise was performed for Effective Tags using the DADA2 module integrated in the QIIME2 software (Version QIIME2-202006). Thereafter, the Effective Tags were subjected to annotation and abundance analysis to reveal the taxa composition of the samples. Here, ASVs with an abundance score < 5 were filtered out. Using a stacked bar plot (created on QIIME2), the taxonomic composition and its relative abundance across samples were visualised. Further, to study the phylogenetic relationship of each ASV and identify differences in the dominant bacterial taxa between 5-aza-treated and control insects, multiple sequence alignment was performed using QIIME2.

#### 4.11.4. Diversity Estimation

Initially, the data underwent rarefaction curve analysis using the modified function “ggrare” (from the ranacapa R package; [71]) to assess sequencing depth adequacy. Subsequently, ASV abundances were normalised by rarefying all samples to the sequencing depth of the sample with the fewest sequences, and no samples were excluded during this process (Figure S2). All subsequent alpha and beta diversity analyses were conducted on the normalised data.

Alpha diversity across the samples was evaluated for diversity, richness, and evenness using seven indices in QIIME2: Observed ASVs, Chao1, Shannon, Simpson, Dominance, Good’s coverage, and Pieloue. Additionally, to assess community composition complexity and evaluate differences between sample groups, beta diversity estimations (based on weighted and unweighted UniFrac distances) were employed. Principal component analysis (PCA) was conducted using the ade4 and ggplot2 packages in R software (Version 3.5.3) to reduce the dimensionality of the original variables for cluster analysis.

Principal coordinate analysis (PCoA) was then performed to obtain principal coordinates and visualise differences among samples in complex multidimensional data. The three-dimensional PCoA results were visualised using the QIIME2 package.

#### 4.11.5. Statistical Analyses

The Adonis and Anosim functions in the QIIME2 software (Version 202006) were used to validate the significance of the differences in community structure between groups. Further, MetaStat and *t*-test analysis (FDR using Benjamini–Hochberg procedure, adjusted *p*-value cut-off < 0.05) were performed on the normalised data for the identification of taxa that differed significantly between the two groups (i.e., 5-aza-treated and control) at each taxonomic level (i.e., phylum, class, order, family, genus, species) using the R software (Version 3.5.3). Next, Linear Discriminant Analysis (LDA; with an LDA score threshold of 4, total sum scaling normalisation, non-parametric factorial Kruskal–Wallis test and pairwise Wilcoxon test (*p*-value cut-off < 0.05)) was conducted to determine the effect size (LEfSe) of each differentially abundant bacterial taxa. Furthermore, bacterial taxa that were consistently present across the entire microbial community were detected based on their relative abundance and prevalence in samples. A heatmap was generated using MicrobiomeAnalyst (Version 2.0, https://www.microbiomeanalyst.ca/; accessed on 20 July 2023) for improved interpretation and visualisation.

Finally, hierarchical clustering analysis was performed (using the “hclust” function in the R package “stat”) to assess overall variation in bacterial taxa composition and abundance. Here, the distance between samples was measured using Jaccard’s index, and hierarchical clustering was performed via Ward’s linkage algorithm. The resulting cluster relationships were visualised as a dendrogram generated using MicrobiomeAnalyst.

#### 4.11.6. Function Prediction

To study the function of the bacterial community present in the samples and find out the different functions of the communities in the different groups, functional annotation analysis was performed using the PICRUSt2 software (Version 2.1.2b). Function prediction was carried out according to *16S rRNA* sequencing data based on the KEGG database. Based on the function annotation and abundance information of samples, the top 35 functions of the bacterial community and their abundance information in each sample were selected to draw the heatmap clustered at different functional levels. Principal component analysis (PCA) dimensionality reduction analysis was conducted for the function abundance scores. Further, to investigate the distribution of functions among samples (groups), the common and unique functions among different samples (groups) were analysed, and the Venn/Flower diagram was drawn for better visualisation. Furthermore, based on function prediction, metabolic pathway reconstruction was carried out. The functional differences between groups (i.e., 5-aza-treated and control) were validated by Student’s *t*-test and pathways that were differentially present in the two groups were identified.

### 4.12. Analysis of the Impact of 5-Azacytidine on the BPH Immune System

#### 4.12.1. RNA Isolation

RNA was isolated from eight insects per treatment (i.e., 5-aza-treated and control insects) using the RNeasy Plus Mini Kit (Qiagen, Germantown, MD, USA). RNA concentrations and purity were assessed using a NanoDrop Spectrophotometer and integrity was verified by agarose gel electrophoresis following standard protocols [67].

#### 4.12.2. Gene Expression Analysis—qRT PCRs

Quantitative real-time PCR (qPCR) was conducted to investigate the impact of azacytidine-mediated disruption of the methylome on the expression levels of immune-related genes in BPH. Each reaction (10 μL) consisted of SYBR Green PCR Master Mix (5 μL; Thermo Fisher Scientific, Waltham, MA, USA), primers (2.5 μM), and cDNA (5 ng). Primer details are provided in Table S11. PCR conditions were as follows: initial denaturation at 94 °C for 5 min, followed by 40 cycles of denaturation at 94 °C for 10 s, and annealing and extension at 60 °C for 30 s.

Melt curve analysis was carried out after 40 cycles to verify reaction specificity. Relative gene expression was then normalised to the expression level of actin gene. Expression data were presented as relative values using the relative standard curve method, and results were analysed with the 2^−ΔΔCt^ method. Each analysis included 3 biological and 3 technical replicates. Further, the data were subjected to an unpaired, two-tailed Student’s *t*-test (*p*-value cut-off < 0.05) for determining the statistical significance of the data.

### 4.13. Analysing the Impact of 5-Aza and Antibiotic Treatment on Lipid Metabolism in BPH

Fat body tissue of BPH was stained with Nile Red (Sigma-Aldrich, St. Louis, MO, USA) to visualise the changes in lipid metabolism in BPH in response to 5-aza and antibiotic treatment. The BOD insects (untreated) served as a control. Briefly, fat bodies from BPH individuals were dissected in 1× phosphate-buffered saline (PBS) buffer containing 5% Triton X100, by carefully removing the internal organs while retaining the cuticle, and adhering fat body tissue under a dissection microscope. The dissected fat body tissue was fixed in 4% paraformaldehyde for 15 min at room temperature and then washed with 1× PBS. Fat bodies were stained with Nile Red staining solution (prepared following the manufacturer’s instructions; 1 μL of Nile Red stock solution (1 mg/mL, in acetone) in 100 μL of PBS) for 1 h at 37 °C, and then washed with 1× PBS. Nile Red-stained lipid droplets were visualised using a Nikon Eclipse Ci fluorescence microscope (Nikon, Tokyo, Japan) using green (488 nm) and red (543 nm) filters. Images were acquired at both wavelengths and captured using a Nikon digital camera DS-Ri2. The analysis was carried out in triplicate.

## Data Availability

The original contributions presented in this study are included in the article/Supplementary Material. NGS datasets generated during this study are available as Sequence Read Archive (SRA) files at NCBI and can be accessed using the accession numbers PRJNA845094 and PRJNA847772. Further inquiries can be directed to the corresponding author.

## Supplementary Materials

The following supporting information can be downloaded at: https://www.mdpi.com/article/10.3390/ijms27167357/s1.
