# Supplementary material for: Microbiome–Epigenome Interplay Impacts Microbial Symbiosis and Stress Adaptations in the Brown Planthopper (Nilaparvata lugens)
Source: Int J Mol Sci. 2026 Aug 17;27(16):7357. doi: 10.3390/ijms27167357 (PMC13512935; doi:10.3390/ijms27167357)
Supplement: Supplementary file 1 [file ijms-27-07357-s001.zip › ijms-4310434-supplementary/Supplementary Data/Supplementary Figures/Fig S1.pdf]

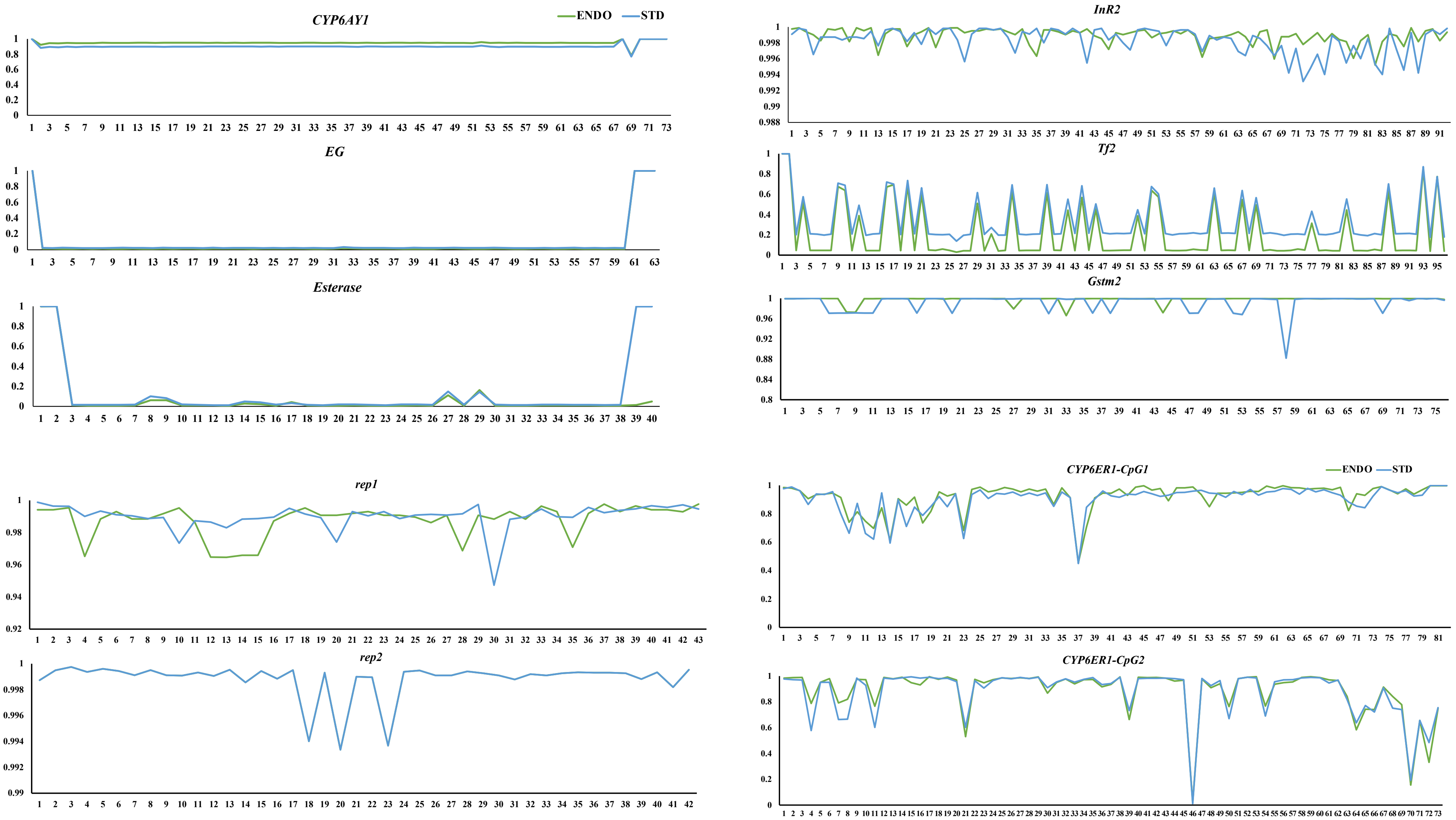

Fig. S1 Site-specific analysis of antibiotics-induced methylation changes across stress-responsive genes in antibiotics-treated (ENDO) and untreated (STD) samples of BPH. Line graphs were plotted for the cytosine sites (within CpG islands corresponding to stress-responsive genes) in all three contexts. Gene names are mentioned on top. Values on the Y-axis represent methylation levels and numbers on the X-axis indicate cytosine position within the CpG island. NOTE: When methylation patterns for a gene are identical or highly similar for both treatments, only a single trend line is visible due to overlap.
