# Supplementary material for: Microbiome–Epigenome Interplay Impacts Microbial Symbiosis and Stress Adaptations in the Brown Planthopper (Nilaparvata lugens)
Source: Int J Mol Sci. 2026 Aug 17;27(16):7357. doi: 10.3390/ijms27167357 (PMC13512935; doi:10.3390/ijms27167357)
Supplement: Supplementary file 1 [file ijms-27-07357-s001.zip › ijms-4310434-supplementary/Supplementary Data/Supplementary Figures/Fig S2.pdf]

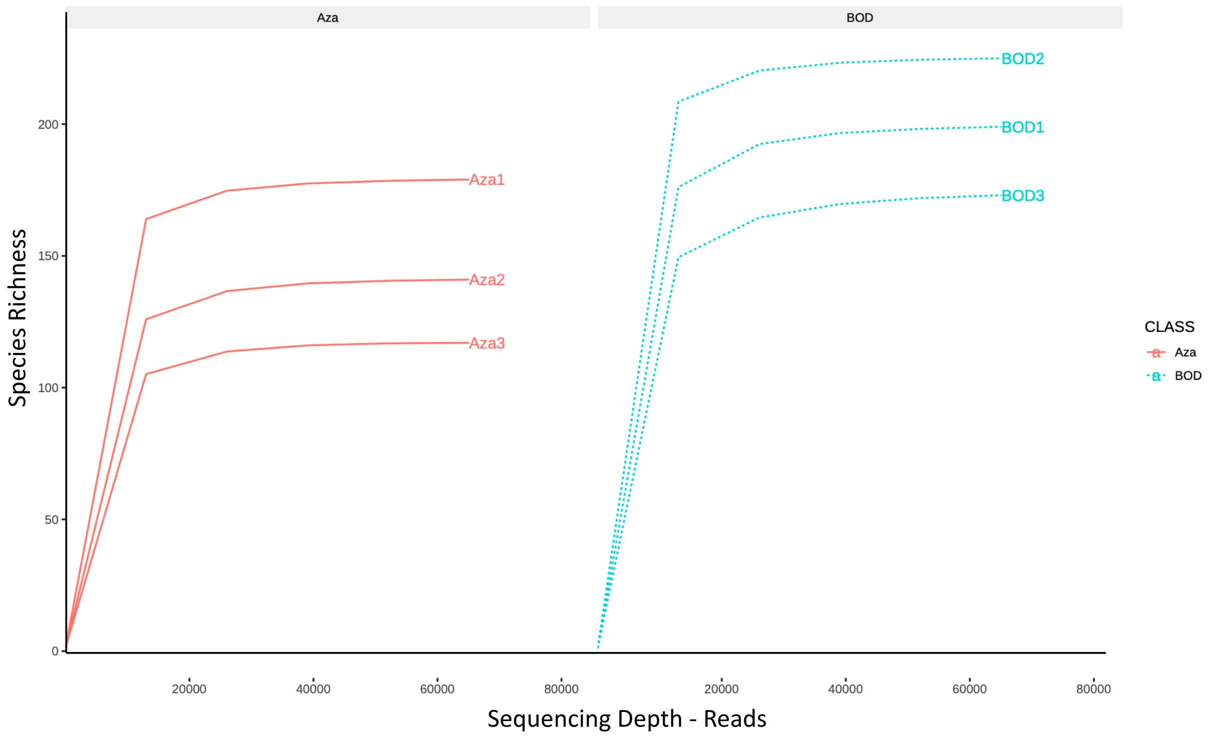

Fig. S2 Comparative rarefaction curves indicating bacterial species richness in 5-aza-treated and untreated (BOD) samples. Numbers on the X-axis indicate sequencing depth and values on the Y-axis represent observed OTUs
