# Supplementary material for: Microbiome–Epigenome Interplay Impacts Microbial Symbiosis and Stress Adaptations in the Brown Planthopper (Nilaparvata lugens)
Source: Int J Mol Sci. 2026 Aug 17;27(16):7357. doi: 10.3390/ijms27167357 (PMC13512935; doi:10.3390/ijms27167357)
Supplement: Supplementary file 1 [file ijms-27-07357-s001.zip › ijms-4310434-supplementary/Supplementary Data/Supplementary Figures/Fig S3.pdf]

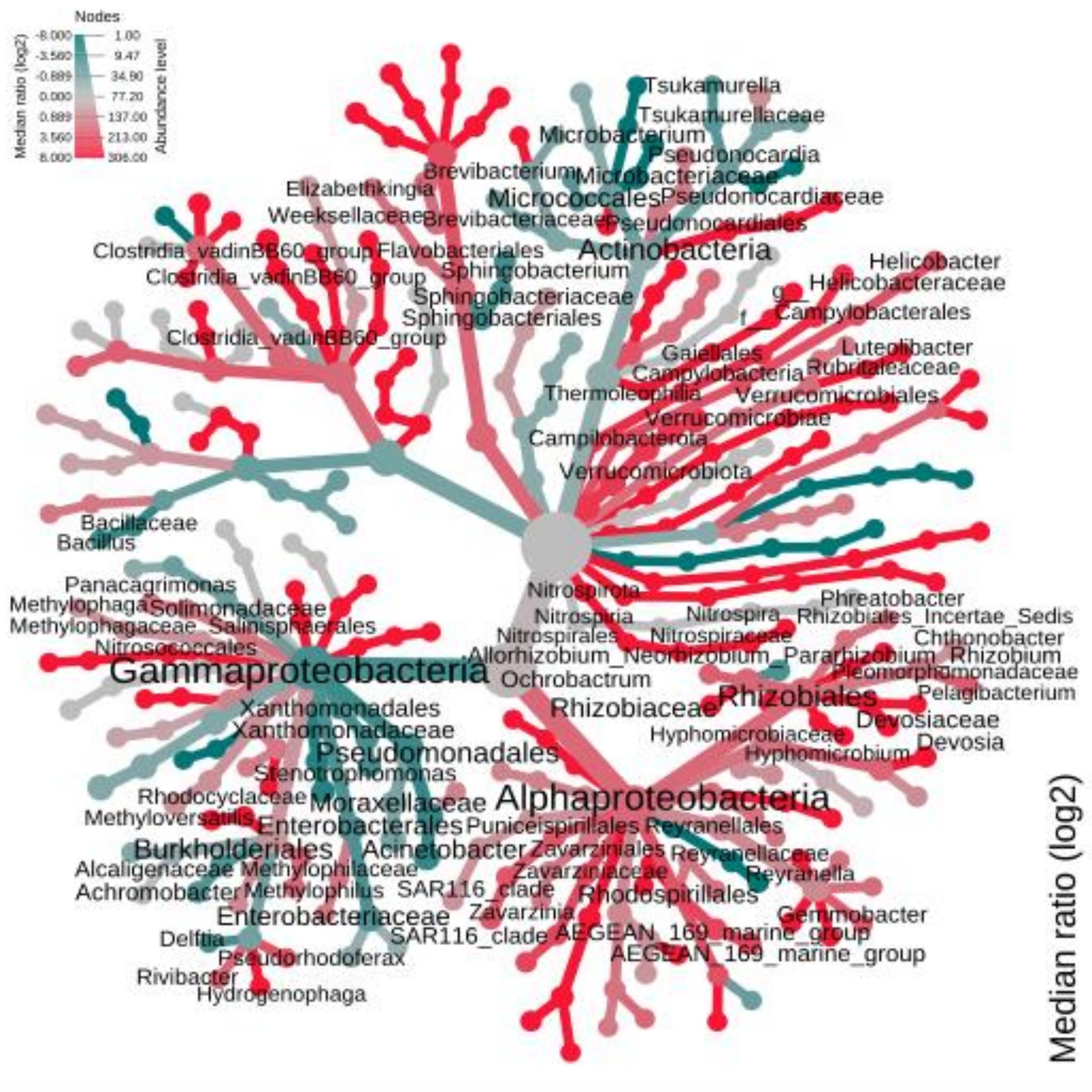

Fig. S3 Differential heat tree depicting the change in taxa abundance between 5-aza-treated (Aza) and untreated (BOD) samples at Family level. The size and colour of nodes and edges are correlated with the abundance of taxa. The size of the individual nodes in the cladogram depicts the number of taxa identified at that taxonomic level. The taxa identified as differently represented are according to the Wilcoxon statistical test. The legend to the nodes can be read as follows: Left side, 'Log2 ratio' of the median proportions of the taxa corresponding to the colour scale, Right side, 'Number of ASVs' where the diameter reflects the number of ASVs corresponding to the nodes on the heat trees. The branches with differential abundance are coloured according to their respective categories shown for each tree. The colour gradient and the size of the node, edge, and label are based on the log2 ratio of median abundance.
