# Supplementary material for: Microbiome–Epigenome Interplay Impacts Microbial Symbiosis and Stress Adaptations in the Brown Planthopper (Nilaparvata lugens)
Source: Int J Mol Sci. 2026 Aug 17;27(16):7357. doi: 10.3390/ijms27167357 (PMC13512935; doi:10.3390/ijms27167357)
Supplement: Supplementary file 1 [file ijms-27-07357-s001.zip › ijms-4310434-supplementary/Supplementary Data/Supplementary Figures/Fig S4.pdf]

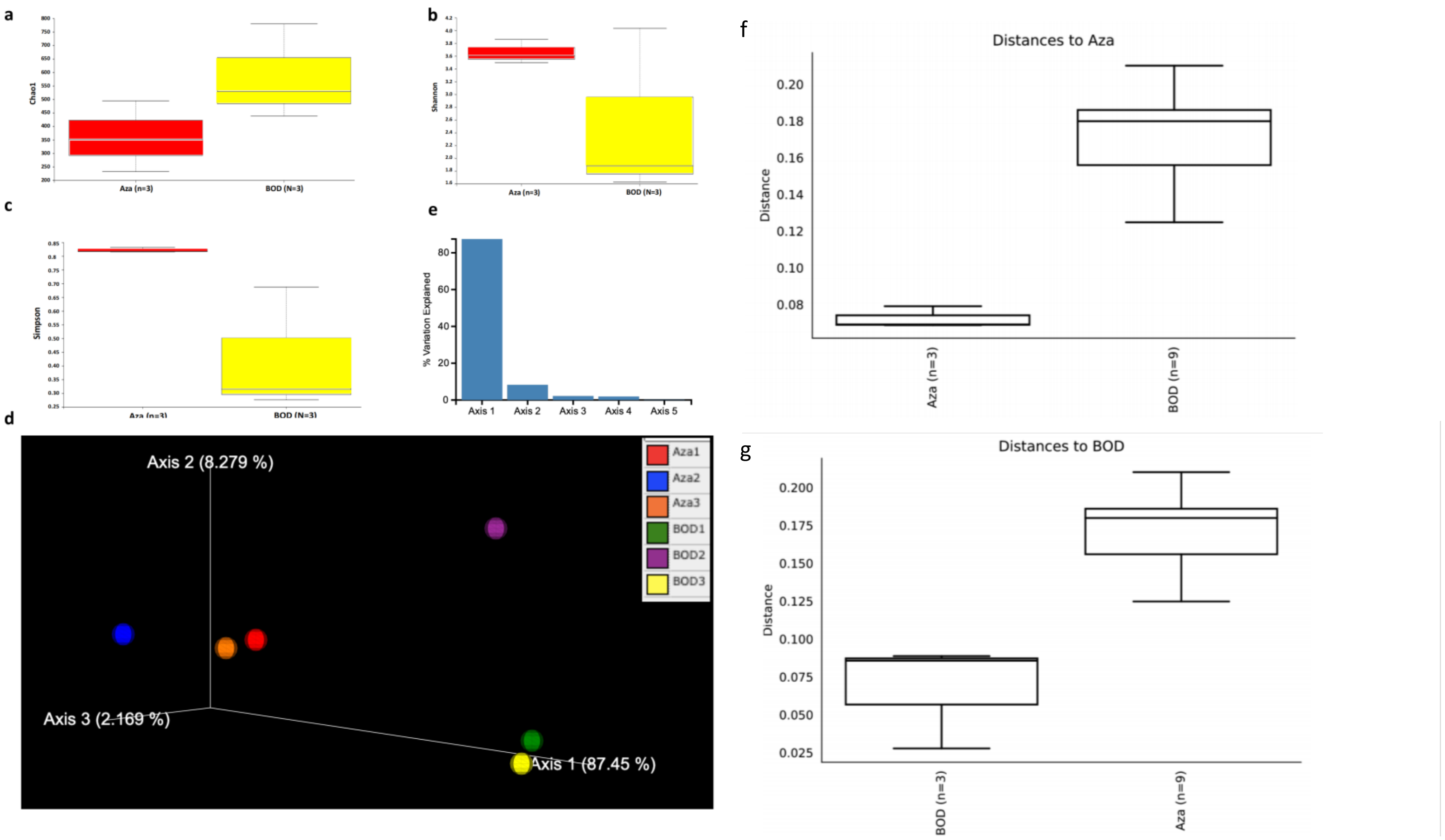

Fig. S4 Bacterial diversity in the control and the 5-aza-treated samples. Box plots represent the alpha diversity distribution of bacterial species in 5-azacytidine treated (Aza) and untreated (BOD) insects. Microbial richness and diversity were measured using a, Chao1, b, Shannon, and c, Simpson indices. d, Beta diversity distribution of bacterial species in the 5-aza-treated (Aza) and untreated (BOD) samples. The 3D principle coordinate analysis (PCoA) plots depicts species diversity and composition with each axis representing variation between samples. e, Scree plot indicating that Principal Component 1 (PC1) captured the most variation followed by PC2 and PC3, and together they accounted for >97% variation between treated and untreated groups. f, Analysis of similarity (ANOSIM) box plots based on weighted Unifrac distances indicating that the differences observed viz. bacterial communities in 5-aza-treated and g, untreated groups, is significant. Boxes indicate interquartile range; horizontal black line indicate the median; box width is proportional to sample size.
