# Supplementary material for: Microbiome–Epigenome Interplay Impacts Microbial Symbiosis and Stress Adaptations in the Brown Planthopper (Nilaparvata lugens)
Source: Int J Mol Sci. 2026 Aug 17;27(16):7357. doi: 10.3390/ijms27167357 (PMC13512935; doi:10.3390/ijms27167357)
Supplement: Supplementary file 1 [file ijms-27-07357-s001.zip › ijms-4310434-supplementary/Supplementary Data/Supplementary Figures/Fig S5.pdf]

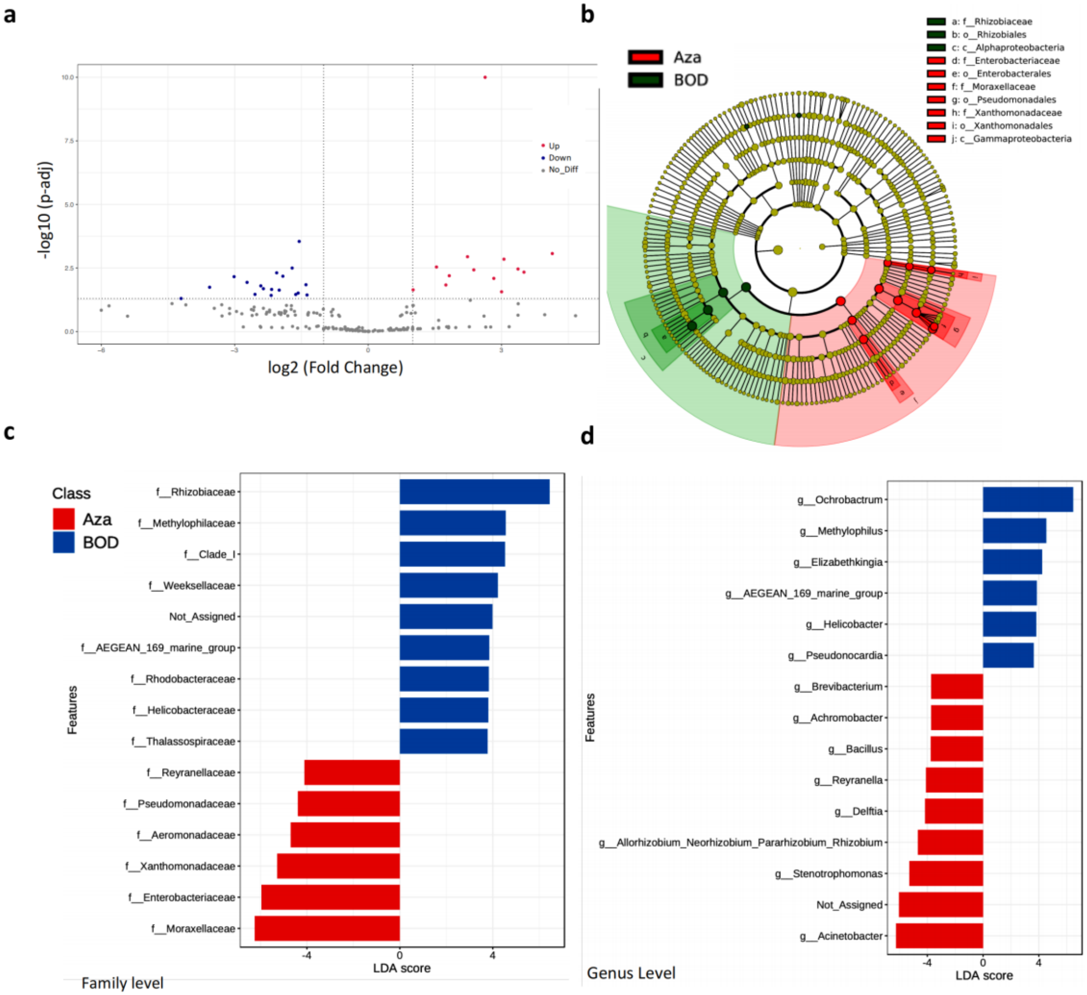

Fig. S5. Variance analysis and significance of differentially occurring bacterial species in the 5-aza-treated and control (BOD) insects. a, Volcano plot highlighting the differentially present bacterial species with fold-change threshold (x) >2 and t-tests threshold (y) < 0.05. The red and blue circles represent features above and below the threshold (dotted line), respectively. The bacterial species showing no significant difference between the two groups are represented by grey circles. Fold changes and p-values are both log transformed. b, Identification of differentially abundant bacterial species between 5-aza-treated (Aza) and untreated (BOD) groups based on Linear discriminant analysis effect size (LEfSe) along with their Linear discriminant analysis (LDA) score. Cladogram plotted from LEfSe analysis showing the taxonomic levels represented by rings with phyla in the outermost the ring and genera in the innermost ring (P < 0.05; LDA score >2). Each circle is a member within that level. Taxa prevalent in 5-aza treated and untreated (BOD) samples are colored red and green, respectively. c, Bar plot representation of bacterial species with LDA score >4 at the family and d, genus levels.
