# Supplementary material for: Microbiome–Epigenome Interplay Impacts Microbial Symbiosis and Stress Adaptations in the Brown Planthopper (Nilaparvata lugens)
Source: Int J Mol Sci. 2026 Aug 17;27(16):7357. doi: 10.3390/ijms27167357 (PMC13512935; doi:10.3390/ijms27167357)
Supplement: Supplementary file 1 [file ijms-27-07357-s001.zip › ijms-4310434-supplementary/Supplementary Data/Supplementary Figures/Fig S6.pdf]

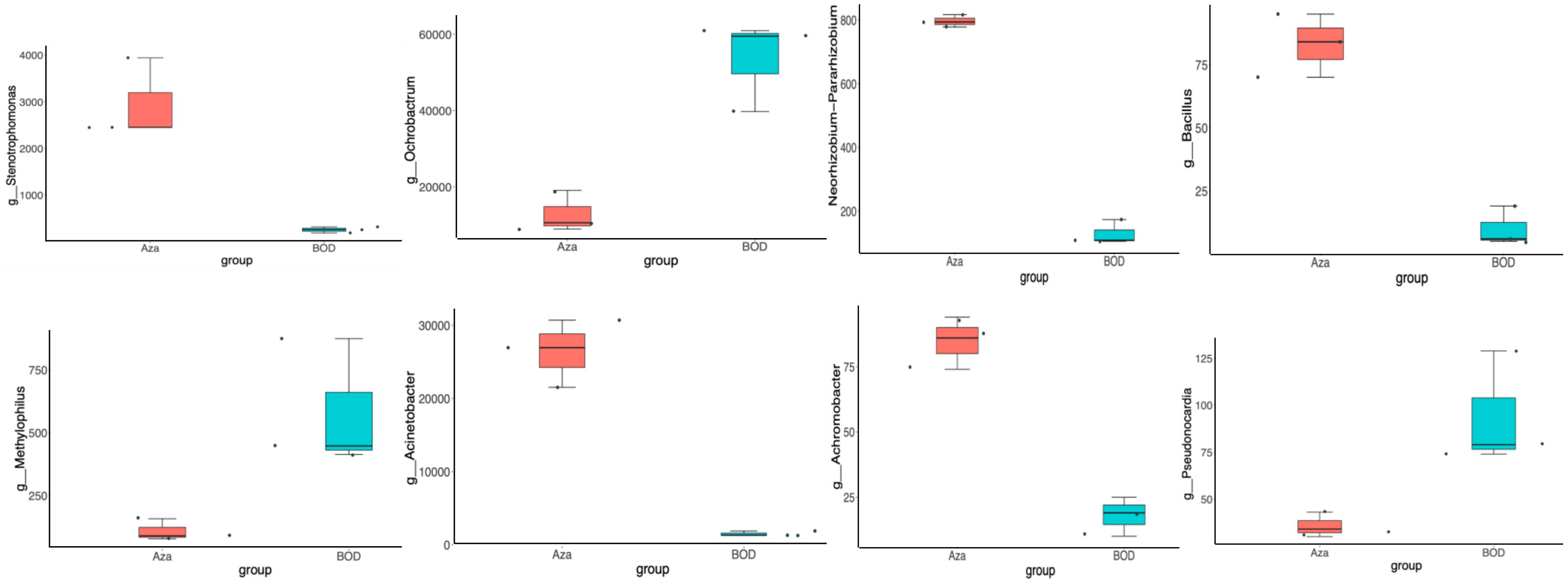

Fig. S6 Box plots depicting the differentially abundant genera that accounted for more than 25% of the *16S rRNA* reads sampled in both populations ($P \leq 0.05$; Aza-treated and BOD samples) as identified by Metastat analysis. Boxes indicate interquartile range; horizontal black line indicate the median; box width is proportional to sample size. Labels on the X-axis represent sample name and numbers on Y-axis indicate bacterial count.
