# Supplementary material for: Microbiome–Epigenome Interplay Impacts Microbial Symbiosis and Stress Adaptations in the Brown Planthopper (Nilaparvata lugens)
Source: Int J Mol Sci. 2026 Aug 17;27(16):7357. doi: 10.3390/ijms27167357 (PMC13512935; doi:10.3390/ijms27167357)
Supplement: Supplementary file 1 [file ijms-27-07357-s001.zip › ijms-4310434-supplementary/Supplementary Data/Supplementary Figures/Fig S7.pdf]

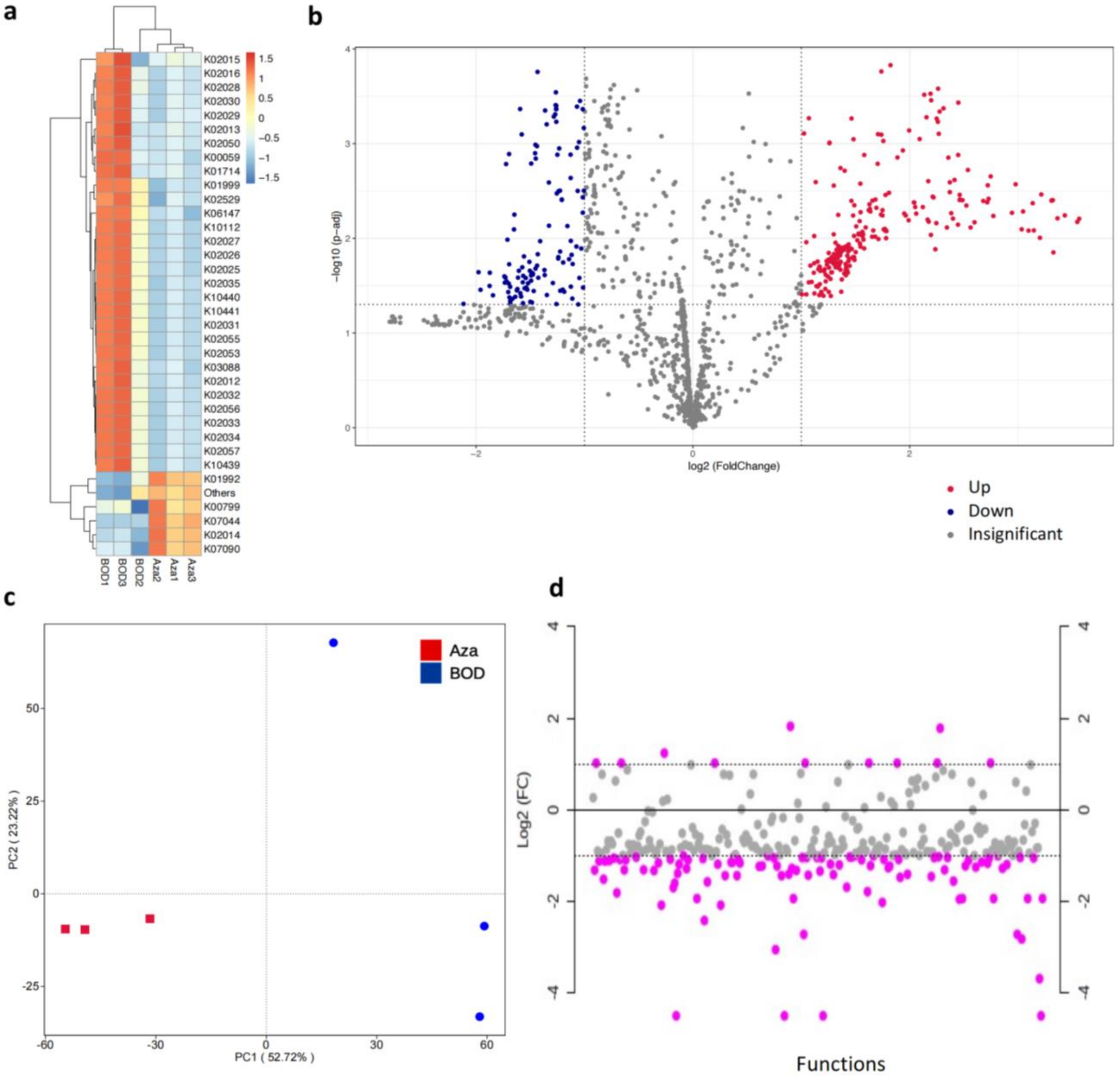


Fig. S7 PICRUSt2 based functional annotation analysis a, Heat map representing functional annotation and their abundance across samples. The top 35 features based on their abundance were plotted and clustered. Sample names and KEGG IDs (representing functional annotations) are indicated on the X-axis and the Y-axis, respectively The clustering was based on functional relatedness of samples; the differences in the relative abundance in the samples are shown by the color gradient where the values increase from blue to red. b, Volcano plot showing functional diversity with reference to the KEGG pathways identified in Aza and BOD samples, with fold change threshold (x) >2 and t-tests threshold (y) < 0.05. The red circles represent features above the threshold and blue dots indicate features below the threshold (horizontal dotted line). The KO categories with no significant difference between the two groups are represented by grey circles. The fold-change and p-values are both log transformed. c, PCA plot representing the clustering of samples based on their functional variability. d, Important pathways selected by fold-change analysis with threshold >2. The pink circles represent features above the threshold and those with no significant difference between the two groups are shown in grey. For the symmetrical presentation of both up- and down-regulated features, the values were log-transformed. The upper and lower thresholds are indicated (dotted lines).
