# Supplementary material for: Microbiome–Epigenome Interplay Impacts Microbial Symbiosis and Stress Adaptations in the Brown Planthopper (Nilaparvata lugens)
Source: Int J Mol Sci. 2026 Aug 17;27(16):7357. doi: 10.3390/ijms27167357 (PMC13512935; doi:10.3390/ijms27167357)
Supplement: Supplementary file 1 [file ijms-27-07357-s001.zip › ijms-4310434-supplementary/Supplementary Data/Supplementary Figures/Fig S8.pdf]

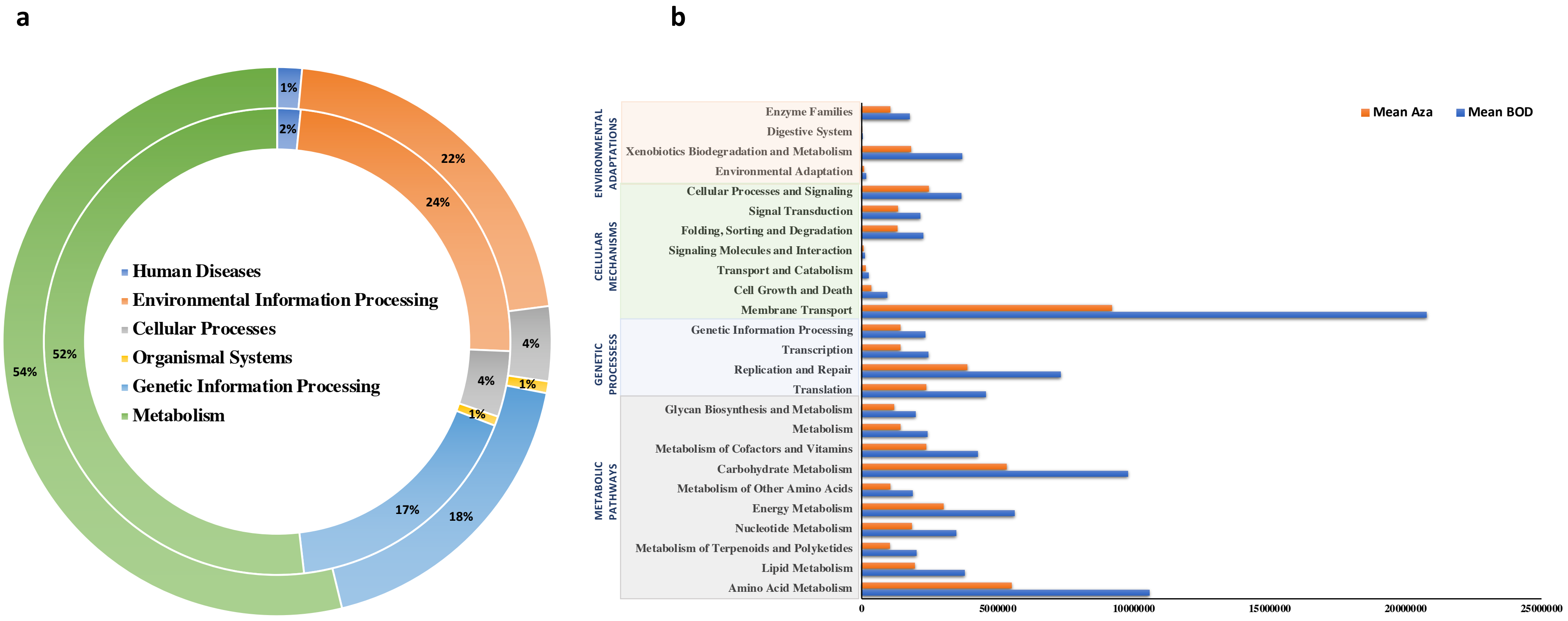

Fig S8. Comparison of PICRUSt-predicted KEGG functions in aza-treated and control samples. a, The contribution of microbial communities present in 5-aza-treated (Aza) and untreated (BOD) samples towards metabolic processes under hierarchy level1 of KO categories. The outer ring and inner rings represent Aza and BOD groups, respectively. b, Significant KEGG categories at hierarchy level2 in 5-aza-treated (Aza) and untreated (BOD) samples along with the corresponding bacterial count/numbers (X-axis) contributing towards each functional pathway.
