# Supplementary material for: Microbiome–Epigenome Interplay Impacts Microbial Symbiosis and Stress Adaptations in the Brown Planthopper (Nilaparvata lugens)
Source: Int J Mol Sci. 2026 Aug 17;27(16):7357. doi: 10.3390/ijms27167357 (PMC13512935; doi:10.3390/ijms27167357)
Supplement: Supplementary file 1 [file ijms-27-07357-s001.zip › ijms-4310434-supplementary/Supplementary Data/Supplementary Figures/Fig S9.pdf]

a **b**

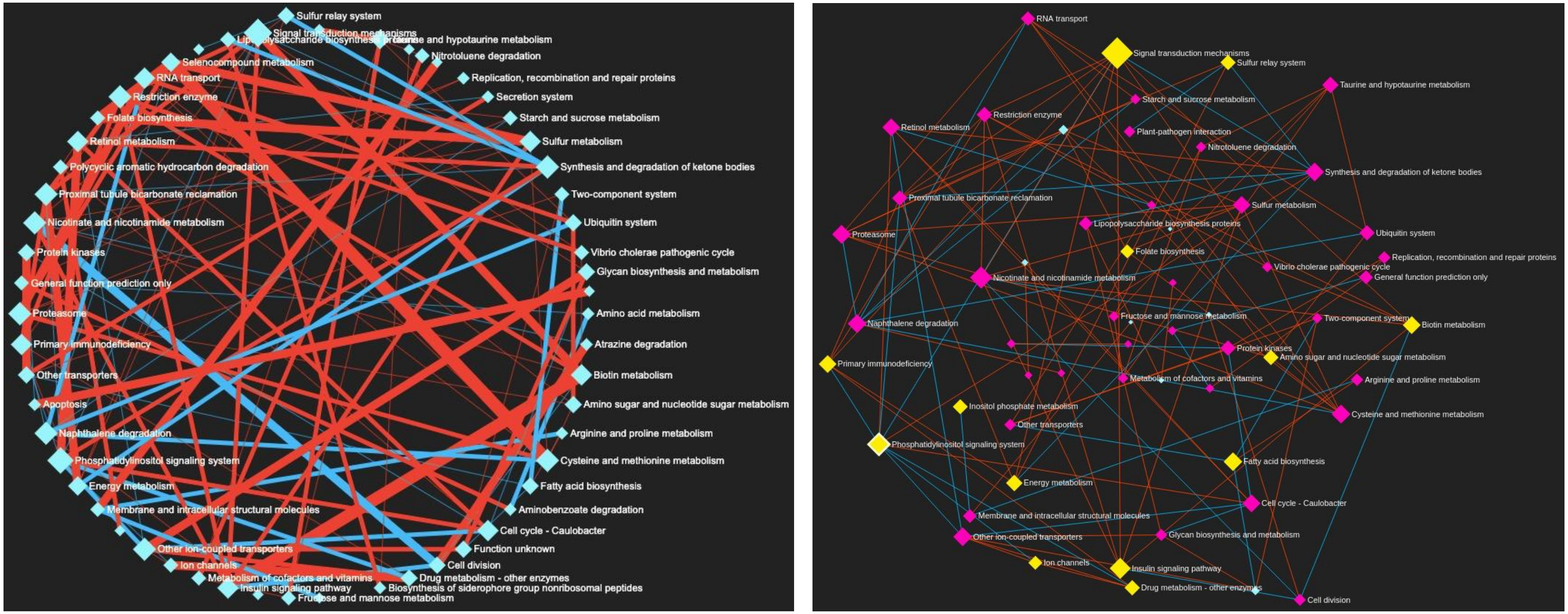

Fig. S9 Differential network enrichment analysis a, Based on debiased sparse partial correlation network modelling revealed various cellular and metabolic pathways that are differentially encoded by Aza and BOD samples (p-value cut off at 0.001). The pathway contained 58 nodes (representing metabolic features) connected with 116 edges (depicting the nature of correlation between them). The positive and negative correlations are represented by red and blue lines, respectively. Edge thickness corresponds to the magnitude of the correlation coefficients. b, Validation and identification of features/nodes crucial to the flow of information within the DSPC network with degree >3 and betweenness >400. Significant nodes (p-value <0.001) are highlighted in yellow.
