# Supplementary material for: Microbiome–Epigenome Interplay Impacts Microbial Symbiosis and Stress Adaptations in the Brown Planthopper (Nilaparvata lugens)
Source: Int J Mol Sci. 2026 Aug 17;27(16):7357. doi: 10.3390/ijms27167357 (PMC13512935; doi:10.3390/ijms27167357)
Supplement: Supplementary file 1 [file ijms-27-07357-s001.zip › ijms-4310434-supplementary/Supplementary Data/Supplementary Tables/Table S1.pdf]

Table S1 Mapping statistics of the targeted bisulfite sequencing reads obtained for each sample.

Sample	PE Reads with quality score > 30	Mapped reads	Mapping efficiency
ENDO	201233	127511	63.36%
STD	219712	73407	33.4%

PE: Paired-end
