# Supplementary material for: Microbiome–Epigenome Interplay Impacts Microbial Symbiosis and Stress Adaptations in the Brown Planthopper (Nilaparvata lugens)
Source: Int J Mol Sci. 2026 Aug 17;27(16):7357. doi: 10.3390/ijms27167357 (PMC13512935; doi:10.3390/ijms27167357)
Supplement: Supplementary file 1 [file ijms-27-07357-s001.zip › ijms-4310434-supplementary/Supplementary Data/Supplementary Tables/Table S11.pdf]

Table S11 Primers used for qRT-PCRs performed in this study.

Primer Name	Primer Sequence (5' – 3')
defensinA F	CAGTTCTTCAGATCCAGTGCGGTCA
defensinA R	CGCTACAGCCGATTCACGACCC
defensinB F	ACTGCTGTTCTTCTGCTGGTTGC
defensinB R	AGCTGCCTCCCTTCTTGCC
PGRP-LB F	ACAATTTTGTGATATGTGGCGATGC
PGRP-LB R	GCTGTTTTGCTAGGTCCAACATCTC
PGRP-LC F	GGACTTCCAGATAGGTAGCGGTAAT
PGRP-LC R	GCTGTAAATGTACCTATGAGAGCAATG
Toll1 F	CGACTTCACCAACAACTTTTTGACG
Toll1 R	TCATGTCGAACCGTGAGATGTTG
Toll7 F	TGCATTATAGGGACCTGCCACACT
Toll7 R	CCCTTCCTCCACCAGCACCA
GRP6 F	CATGCTACGAAGTGCCATCAGC
GRP6 R	CATATTCGACTCCATCCTTCACAACA
GRP4 F	TGAAATCGCACGGTAACAACGAT
GRP4 R	CTGGTAGTCATACGAAGATGGTGC
Proclot1 F	GACATCTGACATTGGGCTTGTG
Proclot1 R	CGTATAGTTGCGATTGCTCAGAC
Snake2 F	ATTCGTGTAGCGGAGATAATGGC
Snake2 R	GTTTGAACGGTGGCGGAGTC
Lysozyme6 F	AGAGTCTGAGGGAGCATACCA
Lysozyme6 R	TCTTCTTCAAACACTCGGGAAACT
